# Relaxation oscillations and hierarchy of feedbacks in MAPK signaling

**DOI:** 10.1038/srep38244

**Published:** 2017-01-03

**Authors:** Marek Kochańczyk, Paweł Kocieniewski, Emilia Kozłowska, Joanna Jaruszewicz-Błońska, Breanne Sparta, Michael Pargett, John G. Albeck, William S. Hlavacek, Tomasz Lipniacki

**Affiliations:** 1 Institute of Fundamental Technological Research, Polish Academy of Sciences, Warsaw, Poland; 2 Institute of Automatic Control, Silesian University of Technology, Gliwice, Poland; 3 Department of Molecular and Cellular Biology, University of California, Davis, California, United States of America; 4 Theoretical Biology and Biophysics Group, Theoretical Division, Los Alamos National Laboratory, Los Alamos, New Mexico, United States of America

## Abstract

We formulated a computational model for a MAPK signaling cascade downstream of the EGF receptor to investigate how interlinked positive and negative feedback loops process EGF signals into ERK pulses of constant amplitude but dose-dependent duration and frequency. A positive feedback loop involving RAS and SOS, which leads to bistability and allows for switch-like responses to inputs, is nested within a negative feedback loop that encompasses RAS and RAF, MEK, and ERK that inhibits SOS via phosphorylation. This negative feedback, operating on a longer time scale, changes switch-like behavior into oscillations having a period of 1 hour or longer. Two auxiliary negative feedback loops, from ERK to MEK and RAF, placed downstream of the positive feedback, shape the temporal ERK activity profile but are dispensable for oscillations. Thus, the positive feedback introduces a hierarchy among negative feedback loops, such that the effect of a negative feedback depends on its position with respect to the positive feedback loop. Furthermore, a combination of the fast positive feedback involving slow-diffusing membrane components with slower negative feedbacks involving faster diffusing cytoplasmic components leads to local excitation/global inhibition dynamics, which allows the MAPK cascade to transmit paracrine EGF signals into spatially non-uniform ERK activity pulses.

A canonical mitogen-activated protein kinase (MAPK) pathway responsible for transducing signals from growth factors consists of three tiers of sequentially activated protein kinases: RAF, MEK, and ERK^1^. Activated ERK, considered the output of the cascade, phosphorylates more than 100 substrates including several transcription factors^2^ and elicits a variety of cellular responses including growth, proliferation, and differentiation^3^,^4^. Unsurprisingly, dysregulated MAPK signaling underlies many cancers^5^. There is growing evidence that the cell fate decisions are regulated by temporal^6^,^7^,^8^ or even spatiotemporal profiles of ERK and RAF^9^,^10^,^11^,^12^,^13^,^14^,^15^. It is thus important to understand how information about the level and gradient of an extracellular stimulus is encoded and transmitted to intracellular downstream effectors.

In the last two decades, numerous positive and negative feedback loops regulating the MAPK network have been discovered and characterized^16^,^17^. A positive feedback from RAS to SOS allows for signal amplification in the vicinity of the receptor and has been proposed to introduce bistability^18^, which enables the system to respond to inputs in a switch-like (digital) fashion^19^. Negative feedbacks emanating from ERK have been associated mainly with response attenuation^20^, but negative feedbacks in general may be harnessed to ensure perfect adaptation^21^,^22^ or give rise to oscillations^23^,^24^,^25^,^26^. System-level mechanisms of controlling information processing in MAPK are still not fully understood, partially due to the fact that systems involving interlocked positive and negative feedback loops may exhibit rich nonlinear dynamical behavior. Dynamics assumed by such complex systems depends on the characteristic time scales involved and network connectivity/topology, i.e., where the feedbacks act and how they relate to each other. For example, sustained oscillations may arise when a negative feedback loop is embedded within a relatively slow positive feedback loop^27^ or when a positive feedback loop is embedded within a relatively slow negative feedback loop^28^. In the latter case, the time profiles may be similar to those produced by a relaxation oscillator, i.e., consisting of a fast activation phase and a phase of (usually slow) relaxation to a state in which subsequent activation is possible^29^. In recent work^30^, we observed similar pulses using a sensor based on phosphorylation-regulated Förster resonance energy transfer (FRET) to monitor EGF-stimulated ERK activity in single MCF10A cells. These pulses differ from constant-frequency quasisinusoidal oscillations in ERK nuclear translocation observed in other studies^26^ and are characterized by an EGF dose-independent amplitude and an EGF dose-dependent period.

In this study, we construct a computational model for MAPK/ERK signaling downstream of EGFR with three aims: (i) to verify whether the combination of the positive and negative feedbacks considered in the model leads to observed relaxation oscillations, (ii) to characterize the functional roles of the different feedback loops depending on their position in the network, and (iii) to analyze the consequences of proteins participating in the feedback loops being localized to distinct subcellular compartments (the plasma membrane or the cytosol) for spatiotemporal profiles of the response. We restrict our analysis to four feedbacks: one positive and three negative (shown in Fig. 1 and discussed in Supplementary Text S1). The proposed model is corroborated by experimental analysis of single-cell responses to a broad range of EGF doses using a fast sensor of ERK activity based on phosphorylation-dependent regulation of nucleocytoplasmic shuttling^31^,^32^.

## Results

### Model

**Figure 1:**
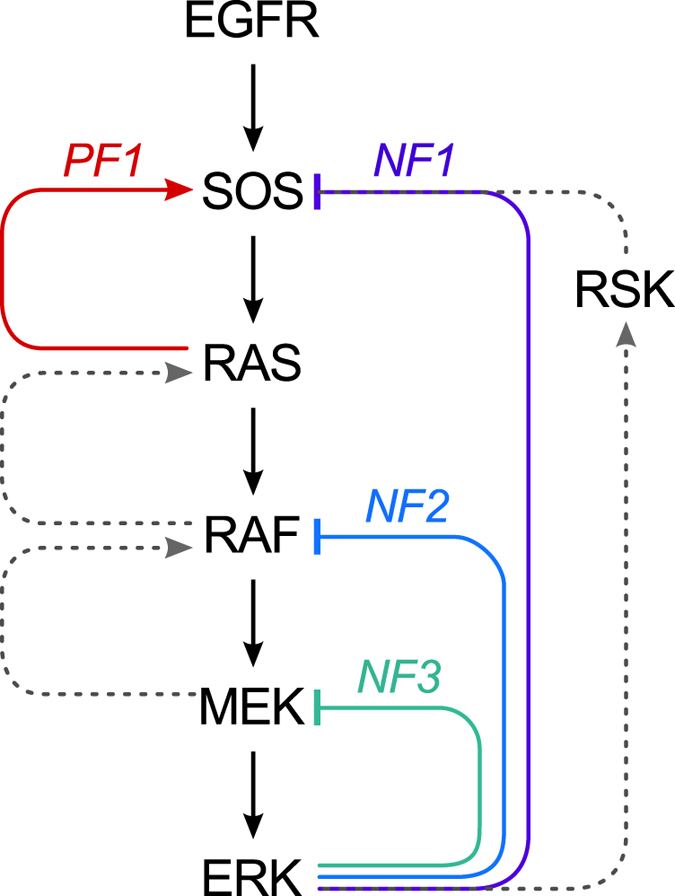
Feedback loops in the MAPK/ERK pathway. Loops captured in the model are shown with solid lines. The arrow labeled PF1 represents positive feedback. This feedback loop has been implicated in mediating bistable switching. The arrows labeled NF1–NF3 represent negative feedbacks. The NF1 feedback loop encompasses the positive feedback loop. The NF2 and NF3 feedback loops are placed out of the positive feedback loop. Dotted lines indicate feedbacks not included in the model.

We constructed a model for EGFR-mediated activation of ERK that captures the feedback loops illustrated in Fig. 1. In addition to well-known negative feedbacks from ERK to its upstream signaling partners, MEK, RAF, and SOS, which have been considered in earlier experimental and modeling studies, our model includes a positive feedback loop involving SOS and RAS. This relatively recently discovered feedback loop, labeled PF1 in Fig. 1, is upstream of negative feedback from ERK to RAF (labeled NF2 in Fig. 1), as well as negative feedback from ERK to MEK (labeled NF3 in Fig. 1), and it is encompassed within the negative feedback loop involving ERK and SOS (labeled NF1 in Fig. 1). It is known that interlocked positive and negative feedback loops can give rise to excitatory behavior, which we have observed in a recent study of single-cell ERK dynamics^30^. Our model was built and analyzed to determine whether positive feedback between plasma membrane-associated signaling proteins could potentially be responsible for the observed behavior, which includes apparent relaxation oscillations in ERK activity.

**Figure 2:**
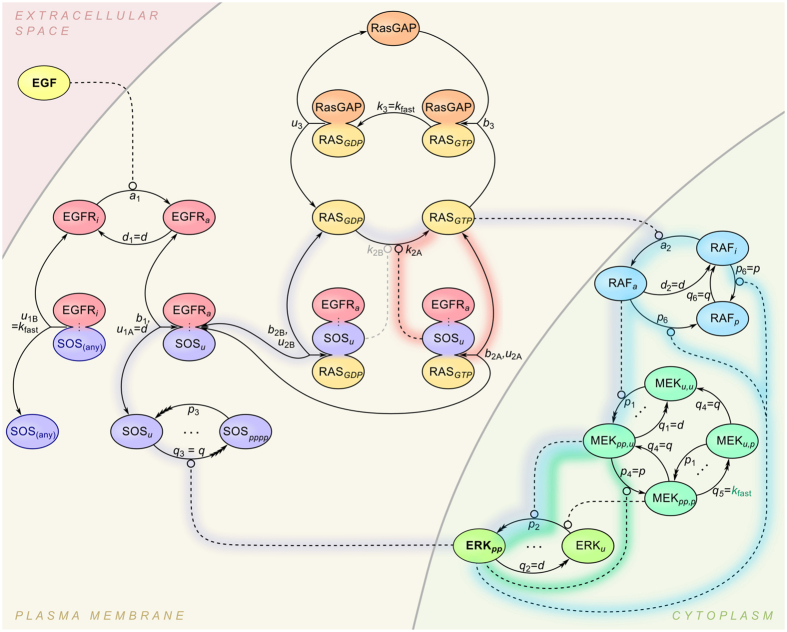
Model for MAPK signaling. Reactions considered in the model are represented by lines with arrowheads. Multiple arrowheads denote reactions with multiple steps (e.g., multi-site phosphorylation of SOS by ERK). Positive influences (e.g., ERK-catalyzed phosphorylation of SOS) are indicated by lines attached to small circles, with the circles identifying the reactions affected. The reactions and influences responsible for various feedback mechanisms are highlighted with shading: RAS-to-SOS positive feedback (red shading), ERK-to-SOS negative feedback (purple shading), ERK-to-MEK negative feedback (green shading), and ERK-to-RAF negative feedback (blue shading). Tripartite arrows, labeled with both binding and unbinding rate constants, are used to represent association and dissociation reactions.

We formulated the model as a system of coupled ordinary differential equations (ODEs) for the mass-action kinetics of the reaction scheme shown in Fig. 2. Although this scheme provides a simplified representation of the MAPK signaling network downstream of EGFR, it preserves the dynamical structure of the network, meaning that it includes the essential processes responsible for the feedbacks connecting the main signaling proteins of the network (cf. Figs 1 and 2). Importantly, with this model, we can assign values to parameters that determine the relative time scales of activating and inhibiting processes. As we will discuss later, we considered two extensions of the model. One is a Markov chain that includes the same processes as those considered in the ODE model but adds stochastic processes that affect the level of EGFR expression on the surface of a single cell. This model extension is intended to account for extrinsic noise and the heterogeneity of cell populations (see Materials and Methods). The second extended model consists of a set of partial differential equations (PDEs). These equations incorporate the right-hand side terms of the ODE model. Reaction–diffusion processes in the extracellular space, on the plasma membrane, and in the cytosol are coupled through Robin-type boundary conditions. This model extension was formulated to study the subcellular spatial heterogeneity of responses to paracrine signaling, modeled as secretion of EGF at random times and random extracellular locations (see Materials and Methods).

An executable specification of the ODE model and the extended stochastic version of the model are provided in Supplementary Data 1. Default parameter settings for the ODE model are summarized in Supplementary Table S1. Supplementary Text S2 and Supplementary Table S2 contain non-dimensional model equations and parameters, respectively. Code for bifurcation analysis are provided in Supplementary Data 2. The stochastic extension of the ODE model has unique parameters that describe the dynamics of EGFR surface expression (Supplementary Table S3) and is provided in Supplementary Data 3. An executable specification of the PDE model is provided in Supplementary Data 4. This model has unique parameters that describe diffusion processes and paracrine signaling (Supplementary Table S4). Parameters were assigned default values as described in Materials and Methods. Simulations and bifurcation analyses were performed as described in Materials and Methods.

### Positive feedback and bistability

**Figure 3:**
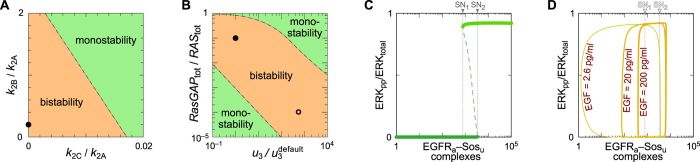
Conditions for bistability and the emergence of relaxation oscillations. With all negative feedbacks removed, the system exhibits either bistable switching or graded responses to EGF stimulation depending on parameter values. (**A**) Range of parameters governing positive feedback for which the system exhibits bistability (for some EGF concentrations higher than 0.1 pg/ml). The parameters are ratios of rate constants, which characterize the relative nucleotide exchange activity of SOS when the REM domain in SOS is not bound to RAS or bound to RAS-GDP compared to the activity when the REM domain is bound to RAS-GTP (*k*_2C_/*k*_2A_ and *k*_2B_/*k*_2A_). We vary these ratios with *k*_2A_ fixed at the value given in Supplementary Table S1. (**B**) Range of parameters governing saturation of RasGAP for which the system exhibits bistability (for some EGF concentrations higher than 0.1 pg/ml). The parameters considered in this diagram characterize RasGAP activity: the ratio of enzyme (RasGAP_tot_) to substrate (RAS_tot_) and the rate constant for dissociation of the enzyme-product complex (*u*_3_). We vary the ratio RasGAP_tot_/RAS_tot_ by varying RasGAP_tot_ while keeping the value of the product *b*_3_ × RasGAP_tot_ constant. This product governs the rate of formation of the enzyme-substrate complex. For more information about parameters, see Supplementary Table S1. In both panels, a solid dot marks the location in parameter space that corresponds to the values of parameters given in Supplementary Table S1. In panel B, an open dot marks the location in parameter space that corresponds to the values of parameters in the model of Stites *et al*. (Ref. 34). (**C**) Bifurcation diagram showing the active ERK fraction vs. the level of EGFR_a_–SOS_u_ complexes in the system without all three negative feedbacks. Solid lines show two stable steady states; dashed line shows unstable steady state. (**D**) Analysis of the system with the negative feedback from ERK to SOS present and other two negative feedbacks absent. Parametric plot shows a projection of the limit cycle on the (active ERK fraction)—(EGFR_a_–SOS_u_ complexes) plane.

According to the model with the default parameter settings of Supplementary Table S1, in the absence of negative feedbacks, the system exhibits bistability, i.e., bistable switching behavior (also known as hysteretic switching). As illustrated in Fig. 3, bistability depends on parameters governing positive feedback between SOS and RAS (Fig. 3A), as well as parameters governing saturation of RasGAP (Fig. 3B).

The results of Fig. 3A are consistent with earlier work, which has implicated positive feedback from SOS to RAS in hysteretic switching^33^. Positive feedback arises because the product of SOS’s GEF activity, which is RAS-GTP, binds to SOS at SOS’s REM domain, which is non-overlapping with SOS’s GEF domain (also known as the Cdc25 homology domain). The outcome of RAS-GTP binding to SOS is an increase in the GEF activity of SOS. RAS-GDP binding to SOS also increases SOS’s GEF activity, but to a lesser extent. The parameter values of Supplementary Table S1 were selected so that positive feedback from SOS to RAS generates hysteretic switching in the absence of negative feedbacks. The parameter values characterizing SOS GEF activity (*k*_2C_, *k*_2B_, and *k*_2A_) are such that SOS’s GEF activity is zero when its REM domain is not bound to RAS (i.e., *k*_2C_ = 0), and its GEF activity when SOS’s REM domain is bound to RAS-GDP is an order of magnitude lower than when SOS’s REM domain is bound to RAS-GTP (i.e., *k*_2B_ = 0.1 × *k*_2A_). As shown in Fig. 3A, bistability requires both parameters, *k*_2B_ and *k*_2C_, to be sufficiently small with respect to *k*_2A_.

Bistability is enabled by constraints on parameters related to SOS’s GEF activity (mentioned above) or by saturability of RasGAP (Fig. 3B). Saturation of RasGAP may occur during a response to EGF if the abundance of RasGAP is sufficiently lower than that of RAS, or if RasGAP-RAS complexes are sufficiently long lived. We have set parameter values such that RasGAP is saturable (Supplementary Table S1). Different parameter settings used in an independent modeling study^34^ are also consistent with saturability of RasGAP (Fig. 3B). In the steady state, the outcome of the pathway depends on the level of EGF-activated and SOS-bound receptors. In the bifurcation diagram of Fig. 3C, the level of EGFR_a_–SOS_u_ complexes serves as a bifurcation parameter. Here, we use the notation “EGFR_a_–SOS_u_” to refer to activated EGFR in complex with SOS, which can bind EGFR only when not phosphorylated. Bistability arises for a certain range of EGFR_a_–SOS_u_ abundance; below this range the system is monostable inactive (i.e., a low fraction of ERK is active), whereas above it, the system is monostable active (i.e., a high fraction of ERK is active).

### Negative feedbacks and oscillations

Analysis of the bifurcation diagram in Fig. 3C suggests that the slow negative feedback mediated by ERK, which reduces the number of EGFR_a_–SOS_u_ complexes (by phosphorylation of SOS to the state in which it cannot bind EGFR), may lead to relaxation oscillations. The mechanism may be explained as follows. In unstimulated cells the level of EGFR_a_–SOS_u_ complexes is below the level corresponding to the saddle node bifurcation point SN_1_ in Fig. 3C, and thus ERK is inactive. EGF stimulation activates receptors, with formation of EGFR_a_–SOS_u_ complexes. When the level of EGFR_a_–SOS_u_ exceeds the level corresponding to the saddle node bifurcation point SN_2_, ERK is activated. Subsequently, ERK phosphorylates SOS, reducing the level of EGFR_a_–SOS_u_ complexes. When this level drops below SN_1_, ERK activity is terminated. After ERK activity is terminated, SOS is dephosphorylated and the system resets; therefore, with persistent EGF stimulation, ERK activity pulses can be recurrent. The cycle is shown in parametric plot in Fig. 3D.

**Figure 4:**
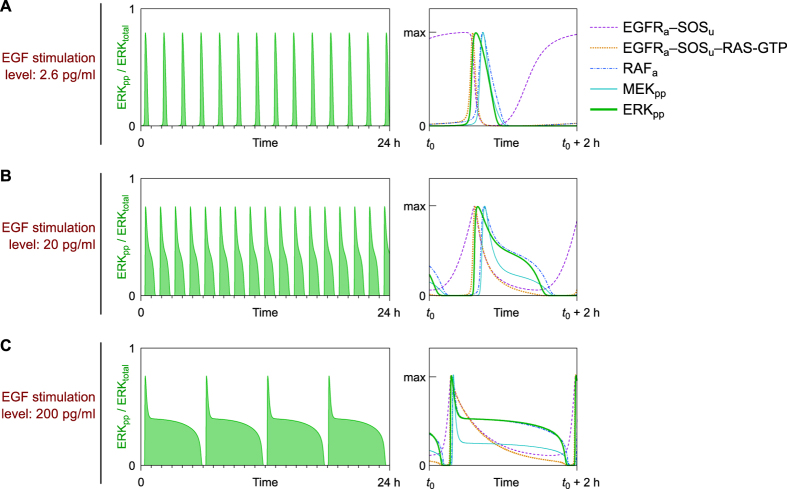
Simulated trajectories for three EGF stimulation doses. The active ERK fraction (left column) together with levels of EGFR_a_–SOS_u_ complexes, EGFR_a_–SOS_u_–RAS-GTP complexes, RAF_a_ and MEK_pp_ normalized to their maxima (right column) are shown as a function of time after stimulation by (**A**) low, (**B**) intermediate, and (**C**) high doses of EGF.

The full system (with all three negative feedbacks illustrated in Fig. 1 and parameterization according to Supplementary Table S1) can exhibit oscillations (Fig. 4). The amplitude of these oscillations very weakly depends on the strength of EGF stimulation, in contrast to the period and pulse width. These properties, indicating that the system switches between on and off states corresponding to the steady states of the system when only the positive feedback is present (Fig. 3C), are characteristic of relaxation oscillations. At a low level of EGF stimulation, ERK activity is pulsatile (Fig. 4A). In contrast, at a high level of EGF stimulation, pulse width is wide and off time is short, such that ERK activity is high most of the time (Fig. 4C). As a consequence, the ERK activity integrated over a population of (unsynchronized) cells would be expected to increase gradually with the EGF dose.

In the analyzed MAPK relaxation oscillator, period of oscillations is controlled by the speed of the inhibition and relaxation processes: inhibition relies on the phosphorylation of SOS by phosphorylated ERK (ERK_pp_), whereas relaxation relies on the resurgence of dephosphorylated SOS (SOS_u_) that can form complex with active EGFR (EGFR_a_) – see Fig. 4, violet dashed line. In the model we assume that SOS phosphorylation and dephosphorylation are possible only when SOS is not bound to EGFR. At a low stimulation dose (Fig. 4a) the resurgence phase is long, because SOS_u_ has to recover to a high level (higher than in the case of a high stimulation dose) in order initiate a next pulse; at a high stimulation dose (Fig. 4c) the inhibition phase is long because SOS_u_ has to drop to a low level (lower than for low stimulation dose) in order to terminate pathway activation.

The prediction of relaxation oscillations (Fig. 4) is consistent with observations of periodic ERK activity made using two different ERK activity reporters, EKAR3 and ERKTR^32^. The ERKTR reporter indicates bursts of ERK activity separated by distinct periods of essentially no activity. The EKAR3 reporter indicates similar oscillatory dynamics, but the off periods are less pronounced (see Fig. 5B in ref. 32). To determine if these features can be explained by the different kinetics of ERK interaction with the two reporters on the top of the model of Fig. 2 we explicitly included ERK activity reporters (see Materials and Methods). As shown in Supplementary Figure S3, the shape of predicted oscillations in the level of reporter phosphorylation depends on the kinetics of ERK-reporter interaction. With slower interaction kinetics, the off periods become shorter.

**Figure 5:**
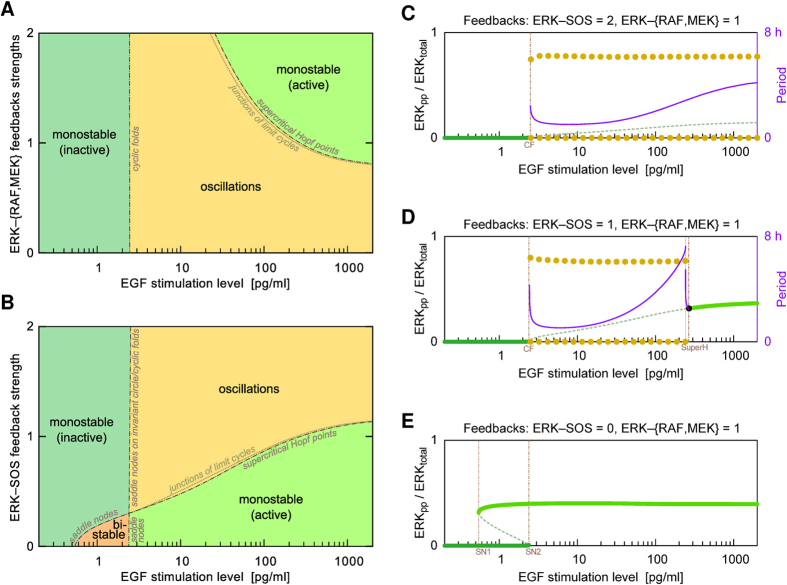
Bifurcation diagrams. Each plot indicates the recurrent solutions for the fraction of activated ERK (ERK_pp_/ERK_total_) as a function of either one or two bifurcation parameters. EGF dose (or stimulation level) is a bifurcation parameter in each plot. In the two-dimensional bifurcation diagrams, the second bifurcation parameter characterizes the strength of the negative feedbacks from ERK to RAF and MEK (**A**) and the strength of the negative feedback from ERK to SOS (B). Areas are colored to indicate distinct qualitative behaviors: oscillatory, monostable with low or high ERK activity, and bistable. The boundaries are labeled to indicate the bifurcation types that separate the different regimes of behavior. The one-dimensional bifurcation diagrams correspond to different strengths of the negative feedback from ERK to SOS (i.e., they correspond to different cross-sections of the parameter space of panel **B**): twice the default strength (**C**), the default strength (**D**), and absent (**E**). Solid green lines indicate stable steady states; dashed green lines indicate unstable steady states. Yellow dots indicate the upper and lower envelopes of stable limit cycles. Purple lines indicate the periods of oscillations. Cyclic fold (CF) bifurcations in panels C and D are accompanied by subcritical Hopf bifurcation points (not marked). In a tiny parameter region between the two bifurcation points there coexist a stable limit cycle, an unstable limit cycle, and a stable steady state. In panel D, supercritical Hopf bifurcation lies close to a series of complex bifurcations (seen as the period discontinuity) which effects in a junction of limit cycles.

To investigate the parameter dependence of oscillatory behavior, we performed a bifurcation analysis (Fig. 5). Two-dimensional bifurcation diagrams (Fig. 5A and B) illustrate how qualitative behavior depends on the strength of EGF stimulation and the strengths of three negative feedbacks. As indicated in Fig. 5B, four regimes of behavior are possible: monostable on and off states, bistability (wherein the steady state occupied depends on history), and oscillations. These regimes are separated by bifurcations of different types, which are indicated by the labeling of boundaries. Figure 5C–E are one-dimensional bifurcation diagrams (with the strength of EGF stimulation chosen as a bifurcation parameter) corresponding to different cross-sections of the parameter space of Fig. 5B. The cross-section in Fig. 5D corresponds to the default parameter values of Supplementary Table S1. This diagram shows that the system is off (on) at low (high) levels of EGF stimulation and exhibits oscillations over a ~100-fold intermediate range of EGF stimulation strength. Over this range, the period of oscillations in ERK activity first decreases and then increases with EGF dose. The default parameters, which were chosen for consistency with observed system behaviors^30^, allow for a rich repertoire of behaviors. Moreover, the relatively modest modification of these parameters can change the system behavior from bistable to oscillatory, which is in line with the conjecture that complex systems are poised at criticality^35^.

Oscillatory behavior depends on feedback strengths (Fig. 5A–D), and bistability is only realized when negative feedback from ERK to SOS is weak or absent (Fig. 5B and E). As can be seen by inspecting Fig. 5B and also by comparing Fig. 5C and D, the range of oscillatory behavior increases with the strength of negative feedback from ERK to SOS. Conversely, the range of oscillatory behavior shrinks with stronger feedbacks from ERK to MEK and RAF (Fig. 5A). Generally, for a system with a single negative feedback, increasing the strength of negative feedback tends to switch off the response of the system to a signal. Here, somewhat paradoxically, increasing the strength of the negative feedbacks from ERK to MEK and from ERK to RAF can push the system from oscillatory behavior to a persistent on state if EGF stimulation is sufficiently strong (Fig. 5A). This behavior arises because negative feedbacks from ERK to MEK and from ERK to RAF weaken ERK activity, such that the ERK-to-SOS negative feedback cannot be engaged to induce oscillations.

We note that oscillatory behavior requires not only a sufficiently strong negative feedback from ERK to SOS (Fig. 5) but also multi-site phosphorylation of SOS (Supplementary Figure S2). In the model, the effect of (distributive) multi-site phosphorylation is to introduce a delay in reactivation of SOS after deactivation of ERK. The delay arises from the time required for phosphatases to act and an assumption that phosphorylation of a single site in SOS by ERK is sufficient to suppress SOS activity. The model accounts for four SOS residues that can be phosphorylated by ERK. If the number of such residues is reduced to three, oscillatory behavior is observed over a much narrower range of EGF concentrations and oscillations are not observed for less than three phosphorylatable residues in SOS (Supplementary Figure S2).

**Figure 6:**
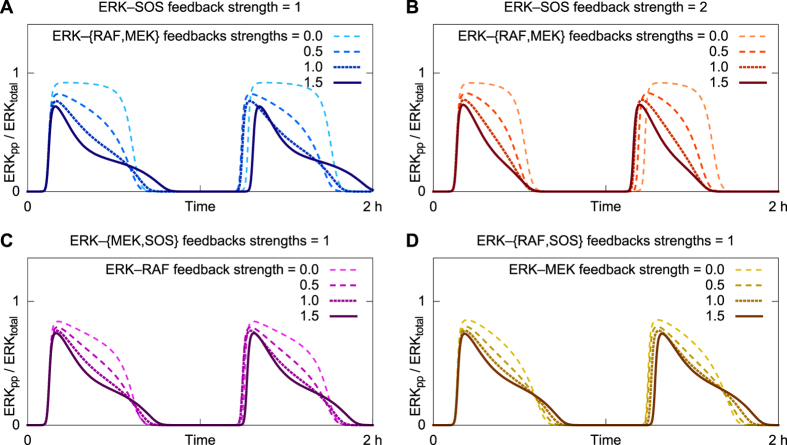
Negative feedback from ERK to MEK and RAF shape the time profile of response to EGF stimulation at 10 pg/ml. Each time course illustrates an oscillatory response to EGF stimulation. In the top panels, the strength of ERK-to-SOS negative feedback is set at the default level (**A**) or twice the default level (**B**). In these panels, different dash patterns correspond to different strengths of feedback from ERK to MEK and RAF, as indicated in the legends. The ERK-to-MEK and ERK-to-RAF feedback strengths are taken to be equal. In the bottom panels, the ERK-to-MEK and ERK-to-RAF feedback strengths are varied separately, as indicated in the legends.

The shape of ERK activity pulses is controlled by the strengths of negative feedbacks from ERK to MEK and from ERK to RAF (Fig. 6). As illustrated in Fig. 6A and B, for a given strength of negative feedback from ERK to SOS, increasing the strengths of negative feedbacks from ERK to MEK and from ERK to RAF changes the waveform of system response from a square wave to a long-tail pulse. Although the ERK-to-MEK and ERK-to-RAF feedbacks are somewhat redundant, both being downstream of positive feedback from RAS to SOS, the ERK-to-RAF feedback is more far ranged and therefore more strongly impacts pulse shape (Fig. 6C and D). Note that we have taken the ERK-to-MEK and ERK-to-RAF feedbacks to be equally strong (Supplementary Table S1). Additional phase space analysis in Supplementary Figure S4 shows in parametric plots that cycle trajectories with two feedback strengths in common group together in tight clusters whereas trajectories with only one feedback strength in common group together in looser clusters.

### Noisy oscillatory excitation of ERK

**Figure 7:**
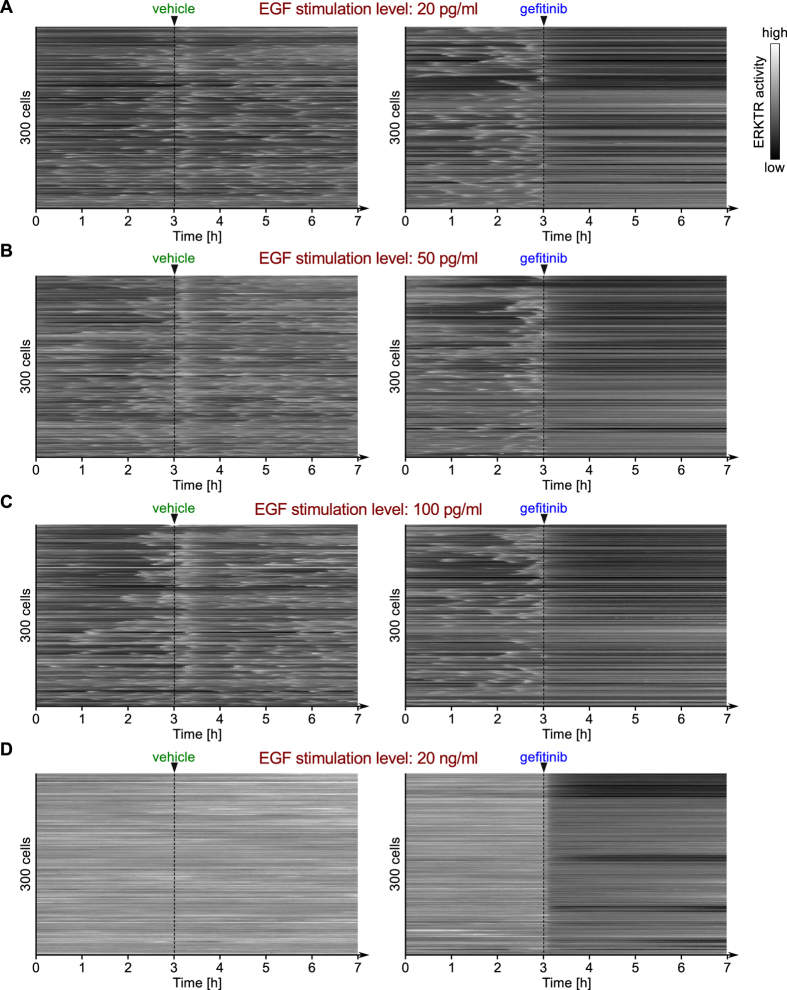
Kinetics of ERK activity as reported by ERKTR at four different doses of EGF. EGFR inhibition by gefitinib is sufficient for elimination of ERK pulses. The time points are spaced by 3 minutes. A smaller set of data from the same experiment was published previously^32^.

In earlier work^30^, using the EKAR-EV reporter, we observed bursts of ERK activity at low EGF doses, regular pulses of ERK activity at intermediate EGF doses, and sustained ERK activity with short, intermittent off periods at high EGF doses. These behaviors have been confirmed using the ERKTR reporter (Ref. 32, Fig. 7), which because of its faster kinetics is able to better resolve oscillatory dynamics (Supplementary Figure S3).

As discussed above, the model of Fig. 2 predicts that oscillations, when they appear, will have an EGF dose-dependent frequency and dose-independent amplitude (Fig. 4). These features of predicted oscillatory behavior in single cells are similar to the oscillations in ERK activity that we observe in single cells (Ref. 30 and Fig. 7). However, the experimentally observed oscillations are noisy, with irregularity both within individual cells and across cells in the population monitored in experiments. To investigate how these irregularities could potentially arise (and whether they are consistent with the model), we extended the model of Fig. 2 to account for extrinsic noise. For simplicity, we considered only a single extrinsic noise source, which we took to be stochastic, time-varying cell-specific surface expression of EGFR. This extension is consistent with heterogeneity in protein expression levels across cell populations, which has been extensively studied and linked to bursts of gene expression^36^,^37^.

**Figure 8:**
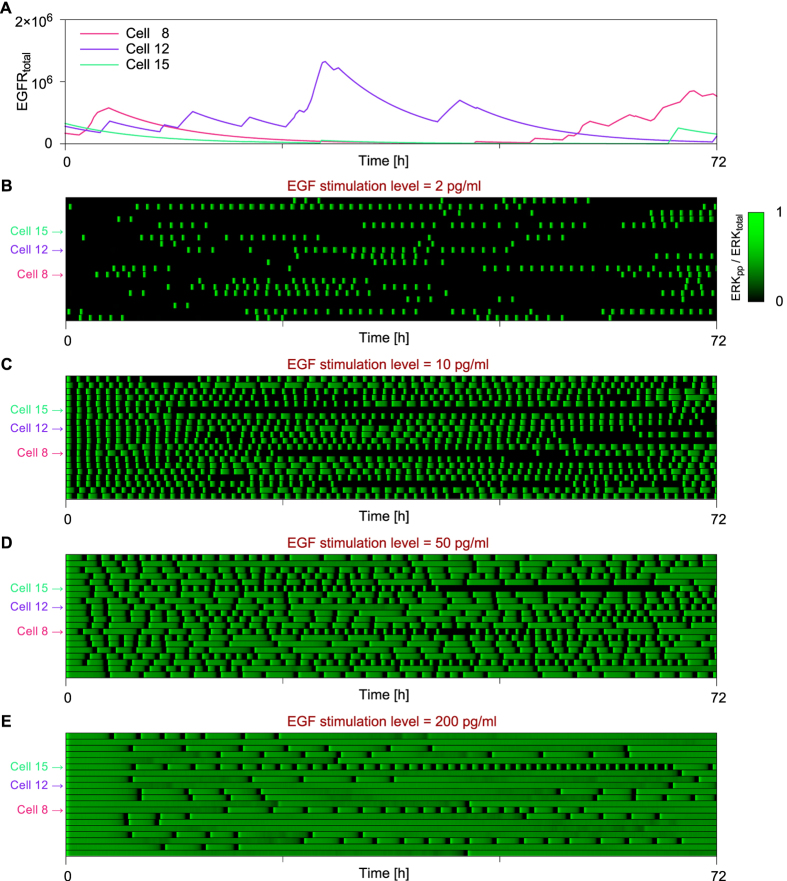
Single-cell temporal EGFR surface expression and ERK activity profiles obtained from stochastic simulations with fluctuating EGFR levels. We considered the effect of extrinsic noise on oscillations in ERK activity. As illustrated in panel (**A**), which shows representative single-cell simulations, we took EGFR surface expression dynamics to be stochastic and marked by bursts of synthesis/expression of variable size (total amount of EGFR added to the plasma membrane) and duration, with bursts being separated by intervals of variable duration. The bottom four panels show heat maps that indicate single-cell responses (ERK activity as a function of time) to different levels of EGF stimulation: (**B**) 2 pg/ml, (**C**) 10 pg/ml, (**D**) 50 pg/ml, and 200 pg/ml (**E**). Each row in a heat map corresponds to an individual cell characterized by a unique set of parameters for EGFR surface expression dynamics. The cells considered are otherwise identical. To demonstrate and isolate the effect of EGF dose, we have taken the temporal EGFR expression profile to be the same across panels for each cell (i.e., each row in each heat map corresponds to a common profile). Supplementary Table S3 provides information about the parameters that are unique for the stochastic simulations shown here.

Thus, the extended model was obtained by recasting the original model as a Markov chain and by adding processes for stochastic generation and clearance of EGFR. Parameters introduced with these extensions are summarized in Supplementary Table S3. Parameter values are such that EGFR surface expression changes on a time scale of hours (Fig. 8A). The influence of extrinsic noise characteristics on active ERK dynamics is analyzed in Supplementary Figure S5. Separate influences of the intrinsic and extrinsic noise on ERK dynamics are demonstrated in Supplementary Figure S6 and Supplementary Figure S7.

Figure 8 shows time courses obtained from the extended, stochastic model for a collection of 20 individual cells responding to different doses of EGF. As can be seen, the extended model predicts irregular EGF dose-dependent excitations of ERK, suggesting that the similar irregularities observed in experiments (Fig. 7) can be attributed to extrinsic noise, which may arise partly from cell-specific time-varying stochastic EGFR surface expression. For the lowest dose considered, 2 pg/ml, ERK is excited in bursts, which tend to be separated by relatively long periods of inactivity (Fig. 8B). According to the deterministic model, this dose lies outside the oscillatory range (Fig. 5D). Indeed, this dose places the system’s steady state in the monostable, inactive regime. The stochastic model predicts bursts of ERK activity arising due to fluctuations of EGFR that allow the level of activated EGFR to exceed a threshold required for ERK activation. We note that inhibition of EGFR (with the kinase inhibitor gefitinib) immediately eliminates pulsatile ERK activity (Fig. 7), indicating that ERK activity pulses require uninhibited EGFR level being above a threshold. Additional analyses of 10-day-long trajectories is provided in Supplementary Figure S8.

### Excitation of ERK by transient EGFR signaling

**Figure 9:**
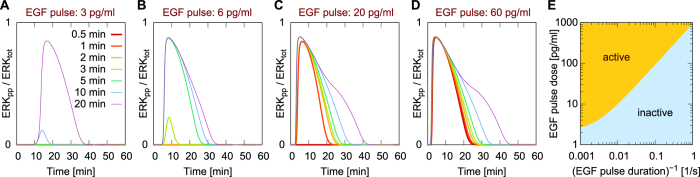
Predicted responses to pulsed EGF stimulations of various doses and durations. Each curve is obtained from a simulation of an experiment wherein EGFR signaling is stimulated by a low (**A**), intermediate (**B,C**) or high (**D**) dose of ligand (EGF) for the duration indicated in the legend. EGF stimulation pulse starts at time = 0. After this pulse of stimulation free ligand is removed. (**E**) Dependence of critical EGF stimulation dose on stimulation duration.

Positive feedback mediated by SOS influences system behavior, such that transient EGF stimulation is expected to produce responses having certain experimentally detectable features. In Fig. 9, we consider predicted responses of the system to different transient doses of EGF. The smallest dose considered, 3 pg/ml, induces a pulse of high ERK activity only for the longest period of EGF stimulation, 20 min (Fig. 9A). In contrast, for a relatively high EGF dose of 60 pg/ml, even the shortest period of EGF stimulation, 30s, induces a pulse of high ERK activity (Fig. 9D). The stimulation time required to induce high ERK activity decreases with increasing EGF dose, which follows from the fact that there exists a threshold level of EGFR that must be activated to trigger downstream signaling. For short EGF stimulation times, active EGFR level increases linearly with time, with a rate proportional to the EGF stimulation dose. Therefore, in this regime, ERK is activated when the product of stimulation time and EGF dose exceeds some threshold. For longer stimulation times, the level of active EGFR saturates; in this limit, the critical dose for long stimulation converges to the critical dose for sustained stimulation, 2.5 pg/ml (cf. Figs 5D and 9E). Figure 9B–D show that for short, transient EGF stimulation periods, ERK activity starts to rise after EGF stimulation stops, which shows that after the level of active EGFR exceeds a threshold, signal propagates independently of further EGF stimulation.

Recently, we observed a similar effect in which the integral of stimulus (i.e., time × amplitude) determines the fraction of activated cells in a population in the case of LPS stimulation^38^. As in the case of growth factor signaling, the immunogenic signal is integrated at the membrane and after surpassing a threshold activates a downstream pathway. This property results from ultrasensitivity or positive feedback operating at the membrane level.

### ERK responses to localized secretion of EGF

A notable feature of the feedback mechanisms involved in MAPK signaling is their ability to propagate signals through space. Positive feedback requires interactions on the plasma membrane (Fig. 2). In contrast, negative feedback signals are relayed by faster diffusing cytosolic proteins (Fig. 2). Such a combination of feedbacks can potentially give rise to local excitation/global inhibition (LEGI) dynamics. In LEGI-type regulation, the membrane components are activated due to propagation of a heteroclinic traveling wave (enabled by bistability introduced by a positive feedback). Activation of membrane components is followed by more uniform activation of cytoplasmic components and then global inhibition of membrane components via negative feedback. LEGI can generate spatial essential for proper cellular responses to directional cues, such as chemotactic movement of ameboid cells toward a higher concentration of a chemokine^39^,^40^,^41^,^42^. Detection of directional cues may be an important function of the EGFR signaling network. EGF gradient sensing or ERK heterogeneities have been implicated in mechanotransduction, cell polarization, and motility^12^,^13^,^14^.

**Figure 10:**
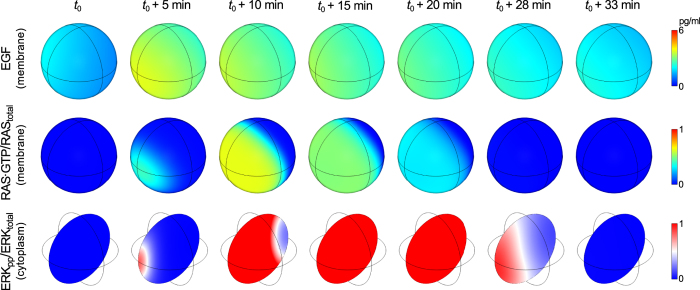
Spatiotemporal dynamics of EGF, RAS activity (RAS-GTP/RAS_total_), and ERK activity (ERK_pp_/ERK_total_) for a cell responding to localized EGF secretion. Shown are sequential snapshots taken from Supplementary Video 1 in the Supporting Information that illustrate the response of a cell to a wave of EGF, which is produced by point sources releasing EGF at random times and locations in a shell surrounding the cell. At each time point, the spatial distribution of EGF at the cell surface is shown in the top row, fraction of activated RAS is shown in the middle row, and the spatial distribution of activated ERK inside the cell is shown in the bottom row (on a cross-section passing through the cell center). Supplementary Table S4 provides information about the parameters that are unique for the spatiotemporal simulations shown here.

To investigate spatial heterogeneities in MAPK signaling, we extended the ODE model of Fig. 2 to obtain a reaction–diffusion PDE-based model. This extended model accounts for both the spatiotemporal kinetics of the signaling network components within the cell and extracellular randomized release of (paracrine) EGF. A more detailed description of this model extension is provided in the Materials and Methods section; parameters introduced with this extension are summarized in Supplementary Table S4. We used the PDE model to predict responses to localized (paracrine) EGF stimulation (Fig. 10). A sequence of simulation snapshots is shown in Fig. 10, which are taken from Supplementary Video 1. This sequence illustrates how non-uniform EGF stimulation, which is visualized in the top row of images, can trigger a traveling wave of activated RAS that spreads over the plasma membrane (Supplementary Video 1) and that produces a transient gradient of ERK activity within the cell, which is visualized in the bottom row of images. This spatiotemporal behavior is in accordance with LEGI dynamics.

Interestingly, ERK may be activated even when the average surface concentration of EGF is below the threshold concentration required for activation in the well-mixed limit (i.e., below the level corresponding to the saddle node bifurcation point SN_2_ in Fig. 5E). This behavior, which can be seen at approximately 20 hr in Supplementary Video 1, occurs because the local concentration of EGF exceeds the average concentration, as well as SN_2_, the threshold required to trigger a traveling wave of RAS activation. Once the heteroclinic wave is initiated on the membrane, its propagation requires only that the concentration of EGF remains above the lower limit of the bistability range, i.e., above the level of EGF corresponding to the saddle node bifurcation point SN_1_ in Fig. 5E.

## Discussion

The MAPK signaling cascade transmits signals that control diverse cell functions such as proliferation, differentiation, motility, and apoptosis^3^. The mechanisms allowing the pathway to achieve specificity are still elusive. There is growing evidence that diverse cellular functions are coordinated through multiple posttranslational modifications of RAF. These modifications allow RAF isoforms to propagate signals into diverse downstream pathways^43^,^44^. Another mode of cell fate control is associated with the temporal profiles of RAF, MEK, and ERK activity. Recently, Ryu *et al*. demonstrated in PC‐12 cells that the choice between proliferation and differentiation depends on the frequency of ERK activity oscillations^8^. Here, we constructed and analyzed a computational model of MAPK signaling to clarify how such oscillations can be generated in response to persistent stimuli, and how their frequency and time profile (i.e., pulse shape) are controlled.

Within the MAPK/ERK signaling cascade, ERK mediates apparently redundant negative feedback loops that inhibit signal propagation at multiple upstream points. In our modeling study, we focused on the positive feedback loop that involves the small GTPase RAS and its guanine nucleotide exchange factor SOS. In the absence of negative feedbacks, this positive feedback loop leads to bistable switching. However, when this loop is nested within the negative feedback loop from ERK to SOS, the system produces relaxation oscillations, which match experimental time courses. The two other negative feedbacks, from ERK to MEK and RAF, play only auxiliary roles in the generation of oscillations, principally modulating the shape of ERK activity pulses. The presence of these two loops, however, allow for controlling activity of RAF, which has numerous targets in addition to MEK^45^. The positive feedback introduces a hierarchy between negative feedbacks, such that the ERK to SOS feedback is responsible for generating oscillations and the other two feedbacks considered in our analysis are responsible for shaping the waveform of oscillations.

In addition to the feedbacks discussed above, there are at least two other positive feedbacks that are nested within the ERK–SOS negative feedback loop: the ERK/MEK hidden feedback, in which bistability may arise in the double phosphorylation/dephosphorylation cycle due to distributive phosphorylation and saturability in dephosphorylation reaction^46^, and the RAS–GAB1–PI3K feedback^47^. Both feedbacks fit into the discussed topology that can give rise to relaxation oscillations, but, importantly, only the RAS–GAB–PI3K feedback (in addition to the RAS–SOS feedback) can act as an initial signal amplifier, because it is upstream of the RAF/MEK/ERK cascade.

Overall, the feedbacks considered here encode a graded input into constant-amplitude pulses, such that the level of a stimulus is translated to the frequency of oscillation and pulse duration. We speculate that, in comparison with amplitude-to-amplitude coding, amplitude-to-frequency coding may decrease ambiguity in signal interpretation by downstream effectors and increase information channel capacity. The NF-κB signaling system, which is characterized by a constant period of oscillations and thus appears to employ the former scheme^48^, was found to transmit only ~1 bit of information^49^,^50^. Interestingly, in the MAPK pathway, Aoki *et al*. found that frequency, but not amplitude, of ERK activity pulses is correlated with cell proliferation rates^51^.

The ERK pathway can modulate cell adhesion and promote cell migration by activation of actin polymerization^52^ or vimentin filament remodeling^53^. To respond properly to extracellular, directional cues^14^,^54^, the pathway should be organized spatially^55^. By analyzing the MAPK/ERK network as a reaction–diffusion system we found that when a cell is exposed to localized bursts of EGF, ERK activation exhibits local excitation/global inhibition (LEGI) dynamics^56^. LEGI dynamics arise because of the existence of a fast positive feedback involving slowly diffusing membrane-bound proteins. Under these conditions, localized EGFR excitation can trigger RAS activation that spreads over the membrane as a traveling wave, which induces a non-uniform surge of RAF, MEK and ERK activity in the cytosol. Ultimately, ERK-mediated phosphorylation of SOS globally inhibits pathway activity because cytosolic proteins diffuse faster than membrane proteins, by at least one order of magnitude. Such a mechanism allows cells to encode information about concentration gradients of extracellular ligand into spatially structured pulses of cytosolic proteins activity. It has been suggested that ERK-controlled EGF shedding can maintain an intrinsic cell spatial polarity^15^ which may be exploited to induce directed cell migration.

In our spatial model, RAS kinetics is simplified: we consider only a membrane pool of RAS and we neglect the existence of membrane microdomains that can be enriched in RAS. We are however aware that the heterogeneity in RAS distribution combined with the positive feedback between RAS and SOS can substantially influence signaling. In our earlier modelling efforts on B-cell activation^57^ we demonstrate that clusterization of membrane components can itself lead to a local activation, which can then spread over the membrane by means of a traveling wave. Also, EGFR and RAS trafficking between plasma membrane and subcellular compartments, and trafficking-dependent signaling from inside the cell (in addition to signaling from the plasma membrane) would influence spatiotemporal activity profiles of downstream components, RAF, MEK, and ERK.

In summary, we have shown that the presence of the short positive feedback coupling RAS and SOS introduces bistability into the system and that this bistability, when frustrated by the negative feedback loop from ERK to SOS, introduces relaxation oscillations which can explain pulsatile time courses of active ERK observed in experiments. This regulatory circuitry allows for translation of graded inputs into constant-amplitude oscillations that have a period and pulse duration determined by signal strength. Because the positive feedback operates on a short time scale and involves slow-diffusing membrane components while the negative feedback operates on a longer time scale but involves faster-diffusing cytoplasmic components, in response to localized EGF stimulation, the system exhibits local activation/global inhibition dynamics. This allows for translation of spatially-graded stimuli to spatially oriented pulses of RAF, MEK and ERK activity, which could be used to direct cell migration or induce polarity.

## Materials and Methods

### Setting parameters

It is challenging to identify parameter values even when abundant experimental data are available to guide parameter estimation^58^. Here, we selected parameter values so as to obtain certain qualitative system behaviors and to allow for qualitative behavior to change in response to variation in the strength of EGF stimulation. This approach is consistent with the hypothesis that cellular regulatory systems operate close to bifurcation points^35^,^59^. First, we considered the system with only the positive feedback loop from RAS to SOS. We set parameter values so that there is a regime of bistability at low EGF stimulation levels. We then adjusted parameter values so that the time required for information to flow from RAS to ERK yields ERK activation kinetics in accord with experimental observations. Then, we considered negative feedback from ERK to SOS. We set parameters influencing this feedback such that SOS inhibition lasts long enough to allow for nearly complete deactivation of RAF, MEK and ERK. Finally, we considered negative feedbacks from ERK to MEK and RAF and set parameter values governing these feedbacks such that the time profiles of ERK activity are sensitive to the feedback strengths.

### Active ERK reporters

The ODE-based model was supplemented with two ERK activity reporters, EKAR3 and ERKTR. Their activation and deactivation reaction rate constants (Supplementary Table S1) have been tuned to be reproduce experimentally measured reporter kinetics^32^.

### Numerical integration of ODEs

The deterministic model is based on an assumption of well-mixed reaction compartments. It was written in terms of reaction rules using the BioNetGen language (BNGL)^60^. The rules, which are provided in Supplementary Data 1, can be processed by BioNetGen^60^ to obtain a reaction network and a corresponding system of coupled ordinary differential equations (ODEs) for the mass-action kinetics of the reaction network. The ODEs were numerically integrated using CVODE^61^, which is an integral component of BioNetGen. We used BioNetGen’s default settings for CVODE parameters, which are appropriate for stiff systems.

### Construction of bifurcation diagrams

Bifurcation diagrams were obtained using Matlab/Matcont^62^. Supplementary Data 2 contains scripts which can be used to obtain the bifurcation diagrams shown in Supplementary Figure S1.

### Modeling stochastic EGFR surface expression dynamics

To investigate how ERK response dynamics are influenced by stochasticity, we introduced a source of extrinsic noise by allowing the EGFR level to change over time and in this way to modulate cellular sensitivity to EGF stimulation. In the stochastic model, EGFR is expressed in bursts and has a predefined half-life, *τ*_EGFR_ (Supplementary Table S3). Bursts occur at random time points, distributed exponentially with a mean waiting time of λ_interburst_^−1^, and have durations that are drawn from an exponential distribution with mean burst duration of λ_burst_^−1^. The rate of EGFR production in each burst is cell-dependent and is computed as the product of a common intensity factor, *A*_burst_, and a cell-specific factor, α_cell_, which is drawn from a log-normal distribution having mean of 1. The simulations are performed using an efficient variation of Gillespie’s direct method^63^ as implemented in BioNetGen^60^. A BioNetGen input file corresponding to the simulations shown for cell 8 in Fig. 8B is provided in Supplementary Data 3.

### PDE simulations

A single cell is considered. It is represented as a sphere of radius *R*_cell_ with volume *V*_cell_. Values of these and others parameters specific to this model extension are summarized in Supplementary Table S4. Membrane and cytosolic proteins diffuse with diffusivities *D*_mem_ and *D*_cyt_, respectively, with *D*_cyt_ = 10 × *D*_mem_. Reaction–diffusion equations on the membrane and in the cytosol are coupled via Robin-type boundary conditions^64^. We assume that the flux Φ of active RAF at the membrane results from its phosphorylation by membrane-tethered RAS-GTP:


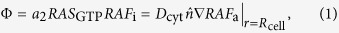


where 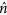 is an outer unit vector normal to the cell surface. The rate of SOS phosphorylation is taken to be proportional to the boundary value of active cytoplasmic ERK. The point sources of EGF appear at random positions in the cell-concentric shell of outer radius *R*_EGF_shell_out_ at random time points throughout the simulation, on average every *f*_EGF_sources_^−1^. EGF releases each have a duration drawn from an exponential distribution with mean duration of λ_EGF_source_^−1^, and a time-independent intensity *A*_EGF_, which is drawn from a log-normal distribution. Released EGF diffuses with diffusivity *D*_EGF_ in the extracellular 3-D space, which is assumed to be unbounded and devoid of obstacles. EGF undergoes degradation with rate constant γ_EGF_ according to the diffusion–degradation equation:


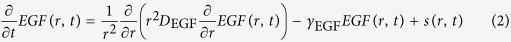


where *r* is the distance from the point source localized at *r* = 0.


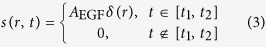


*EGF*(*r, t*) can be calculated using a Green’s function (specific for the diffusion-degradation problem in 3D):


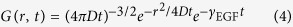


as


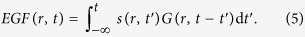


Because


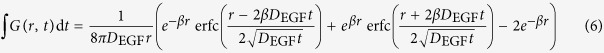


where 
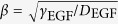
 and erfc is the complementary error function, the contribution from a single EGF source *s(r, t)* equals:


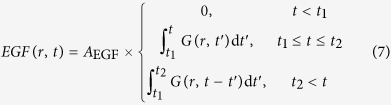


PDE-based simulations were performed using Comsol Multiphysics (see Supplementary Data 4). The spatiotemporal evolution of EGF concentration was evaluated as described above, while reaction–diffusion equations for the cell surface and within the cytosol were solved numerically using a finite element method. For simplicity, a nuclear compartment or other intracellular obstacles were not considered.

### Materials and experimental protocols

Time-lapse imaging of ERK reporter activity was performed as previously described^32^. Briefly, MCF-10A cells stably expressing EKAR3-nes (containing a nuclear export sequence to maintain cytosolic localization) and ERKTR-mCherry were plated on #1.5 glass-bottom multi-well plates coated with type I collagen (BD Biosciences). During imaging, cells were cultured in customized low-fluorescence DMEM/F-12 medium in absence of serum and insulin and the presence of the indicated concentrations of recombinant EGF (PeproTech). At the indicated times, 1 μM gefitinib (Selleck) was added to the medium. Images were collected at 6-minute intervals, and custom MATLAB-based algorithms were used to segment images based on the cytosolic localization of EKAR3 in the YFP channel and extract fluorescence intensities; u-track^65^ was used for cell tracking. ERKTR activity is shown as the ratio of cytosolic to nuclear fluorescence in the mCherry channel.

## Additional Information

**How to cite this article**: Kochańczyk, M. *et al*. Relaxation oscillations and hierarchy of feedbacks in MAPK signaling. *Sci. Rep.* **7**, 38244; doi: 10.1038/srep38244 (2017).

**Publisher's note:** Springer Nature remains neutral with regard to jurisdictional claims in published maps and institutional affiliations.

## Supplementary Material

Supplementary Information

Supplementary Dataset 1

Supplementary Dataset 2

Supplementary Dataset 3

Supplementary Dataset 4

Supplementary video

## References

[b1] Chang F. . Signal transduction mediated by the Ras/Raf/MEK/ERK pathway from cytokine receptors to transcription factors: potential targeting for therapeutic intervention. Leukemia 17, 1263–1293 (2003). 12835716 10.1038/sj.leu.2402945

[b2] Roskoski R. Jr. ERK1/2 MAP kinases: Structure, function, and regulation. Pharmacol. Res. 66, 105–143 (2012). 22569528 10.1016/j.phrs.2012.04.005

[b3] Schaeffer H. J. & Weber M. J. Mitogen-Activated Protein Kinases: Specific Messages from Ubiquitous Messengers. Mol. Cell. Biol. 19, 2435–2444 (1999). 10082509 10.1128/mcb.19.4.2435 PMC84036

[b4] Shaul Y. D. & Seger R. The MEK/ERK cascade: From signaling specificity to diverse functions. *Mitogen-Act*. Protein Kinases New Insights Regul. Funct. Role Hum. Dis. 1773, 1213–1226 (2007). 10.1016/j.bbamcr.2006.10.005 17112607

[b5] Samatar A. A. & Poulikakos P. I. Targeting RAS-ERK signalling in cancer: promises and challenges. Nat Rev Drug Discov 13, 928–942 (2014). 25435214 10.1038/nrd4281

[b6] Murphy L. O., Smith S., Chen R.-H., Fingar D. C. & Blenis J. Molecular interpretation of ERK signal duration by immediate early gene products. Nat Cell Biol 4, 556–564 (2002). 12134156 10.1038/ncb822

[b7] Von Kriegsheim A. . Cell fate decisions are specified by the dynamic ERK interactome. Nat Cell Biol 11, 1458–1464 (2009). 19935650 10.1038/ncb1994 PMC3839079

[b8] Ryu H. . Frequency modulation of ERK activation dynamics rewires cell fate. Mol. Syst. Biol. 11, 838 (2015). 26613961 10.15252/msb.20156458 PMC4670727

[b9] Brown G. C. & Kholodenko B. N. Spatial gradients of cellular phospho-proteins. FEBS Lett. 457, 452–454 (1999). 10471827 10.1016/s0014-5793(99)01058-3

[b10] Kholodenko B. N. Spatially distributed cell signalling. FEBS Lett. 583, 4006–4012 (2009). 19800332 10.1016/j.febslet.2009.09.045 PMC2795127

[b11] Zhao Q., Yi M. & Liu Y. Spatial distribution and dose–response relationship for different operation modes in a reaction–diffusion model of the MAPK cascade. Phys. Biol. 8, 55004 (2011). 10.1088/1478-3975/8/5/055004 21832801

[b12] Mendoza M. C. . ERK-MAPK Drives Lamellipodia Protrusion by Activating the WAVE2 Regulatory Complex. Mol. Cell 41, 661–671 (2011). 21419341 10.1016/j.molcel.2011.02.031 PMC3078620

[b13] Mendoza M. C., Vilela M., Juarez J. E., Blenis J. & Danuser G. ERK reinforces actin polymerization to power persistent edge protrusion during motility. Sci. Signal. 8, ra47 (2015). 25990957 10.1126/scisignal.aaa8859 PMC4830495

[b14] Tschumperlin D. J. . Mechanotransduction through growth-factor shedding into the extracellular space. Nature 429, 83–86 (2004). 15103386 10.1038/nature02543 PMC5539413

[b15] Maly I. V., Steven Wiley H. & Lauffenburger D. A. Self-Organization of Polarized Cell Signaling via Autocrine Circuits: Computational Model Analysis. Biophys. J. 86, 10–22 (2004). 14695245 10.1016/s0006-3495(04)74079-5 PMC1303773

[b16] Shin S.-Y. . Positive- and negative-feedback regulations coordinate the dynamic behavior of the Ras-Raf-MEK-ERK signal transduction pathway. J. Cell Sci. 122, 425–435 (2009). 19158341 10.1242/jcs.036319

[b17] Avraham R. & Yarden Y. Feedback regulation of EGFR signalling: decision making by early and delayed loops. Nat Rev Mol Cell Biol 12, 104–117 (2011). 21252999 10.1038/nrm3048

[b18] Das J., Kardar M. & Chakraborty A. K. Positive feedback regulation results in spatial clustering and fast spreading of active signaling molecules on a cell membrane. J. Chem. Phys. 130 (2009). 10.1063/1.3149861 PMC2832610 19566183

[b19] Xiong W. & Ferrell J. E. A positive-feedback-based bistable ‘memory module’ that governs a cell fate decision. Nature 426, 460–465 (2003). 14647386 10.1038/nature02089

[b20] Cirit M., Wang C.-C. & Haugh J. M. Systematic Quantification of Negative Feedback Mechanisms in the Extracellular Signal-regulated Kinase (ERK) Signaling Network. J. Biol. Chem. 285, 36736–36744 (2010). 20847054 10.1074/jbc.M110.148759 PMC2978602

[b21] Barkai N. & Leibler S. Robustness in simple biochemical networks. Nature 387, 913–917 (1997). 9202124 10.1038/43199

[b22] Alon U., Surette M. G., Barkai N. & Leibler S. Robustness in bacterial chemotaxis. Nature 397, 168–171 (1999). 9923680 10.1038/16483

[b23] Nelson D. E. . Oscillations in NF-κB Signaling Control the Dynamics of Gene Expression. Science 306, 704–708 (2004). 15499023 10.1126/science.1099962

[b24] Geva-Zatorsky N. . Oscillations and variability in the p53 system. Mol. Syst. Biol. 2, 2006.0033 (2006). 10.1038/msb4100068 PMC1681500 16773083

[b25] Kholodenko B. N. Negative feedback and ultrasensitivity can bring about oscillations in the mitogen-activated protein kinase cascades. Eur. J. Biochem. 267, 1583–1588 (2000). 10712587 10.1046/j.1432-1327.2000.01197.x

[b26] Shankaran H. . Rapid and sustained nuclear–cytoplasmic ERK oscillations induced by epidermal growth factor. Mol. Syst. Biol. 5 (2009). 10.1038/msb.2009.90 PMC2824491 19953086

[b27] Pękalski J. . Spontaneous NF-κB activation by autocrine TNFα signaling: a computational analysis. PLOS One 8, e78887 (2013). 24324544 10.1371/journal.pone.0078887 PMC3855823

[b28] Szymańska P., Martin K. R., MacKeigan J. P., Hlavacek W. S. & Lipniacki T. Computational analysis of an autophagy/translation switch based on mutual inhibition of MTORC1 and ULK1. PLOS One 10, e0116550 (2015). 25761126 10.1371/journal.pone.0116550 PMC4356596

[b29] Krishna S., Semsey S. & Jensen M. H. Frustrated bistability as a means to engineer oscillations in biological systems. Phys. Biol. 6, 36009 (2009). 10.1088/1478-3975/6/3/036009 19461130

[b30] Albeck J. G., Mills G. B. & Brugge J. S. Frequency-Modulated Pulses of ERK Activity Transmit Quantitative Proliferation Signals. Mol. Cell 49, 249–261 (2013). 23219535 10.1016/j.molcel.2012.11.002 PMC4151532

[b31] Regot S., Hughey J. J., Bajar B. T., Carrasco S. & Covert M. W. High-Sensitivity Measurements of Multiple Kinase Activities in Live Single Cells. Cell 157, 1724–1734 (2014). 24949979 10.1016/j.cell.2014.04.039 PMC4097317

[b32] Sparta B. . Receptor Level Mechanisms Are Required for Epidermal Growth Factor (EGF)-stimulated Extracellular Signal-regulated Kinase (ERK) Activity Pulses. J. Biol. Chem. 290, 24784–24792 (2015). 26304118 10.1074/jbc.M115.662247 PMC4598990

[b33] Das J. . Digital Signaling and Hysteresis Characterize Ras Activation in Lymphoid Cells. Cell 136, 337–351 (2009). 19167334 10.1016/j.cell.2008.11.051 PMC2662698

[b34] Stites E. C., Trampont P. C., Ma Z. & Ravichandran K. S. Network Analysis of Oncogenic Ras Activation in Cancer. Science 318, 463–467 (2007). 10.1126/science.1144642 17947584

[b35] Mora T. & Bialek W. Are Biological Systems Poised at Criticality? J. Stat. Phys. 144, 268–302 (2011).

[b36] Raj A., Peskin C. S., Tranchina D., Vargas D. Y. & Tyagi S. Stochastic mRNA Synthesis in Mammalian Cells. PLoS Biol 4, e309 (2006). 17048983 10.1371/journal.pbio.0040309 PMC1563489

[b37] Dey S. S., Foley J. E., Limsirichai P., Schaffer D. V. & Arkin A. P. Orthogonal control of expression mean and variance by epigenetic features at different genomic loci. Mol. Syst. Biol. 11, 806 (2015). 25943345 10.15252/msb.20145704 PMC4461400

[b38] Kellogg R. A., Tian C., Lipniacki T., Quake S. R. & Tay S. Digital signaling decouples activation probability and population heterogeneity. eLife 4, e08931 (2015). 26488364 10.7554/eLife.08931 PMC4608393

[b39] Parent C. A. & Devreotes P. N. A Cell’s Sense of Direction. Science 284, 765–770 (1999). 10221901 10.1126/science.284.5415.765

[b40] Xiong Y., Huang C.-H., Iglesias P. A. & Devreotes P. N. Cells navigate with a local-excitation, global-inhibition-biased excitable network. Proc. Natl. Acad. Sci. USA 107, 17079–17086 (2010). 20864631 10.1073/pnas.1011271107 PMC2951443

[b41] Shi C., Huang C.-H., Devreotes P. N. & Iglesias P. A. Interaction of Motility, Directional Sensing, and Polarity Modules Recreates the Behaviors of Chemotaxing Cells. PLoS Comput Biol 9, e1003122 (2013). 23861660 10.1371/journal.pcbi.1003122 PMC3701696

[b42] Bhowmik A., Rappel W.-J. & Levine H. Excitable waves and direction-sensing in Dictyostelium discoideum : steps towards a chemotaxis model. Phys. Biol. 13, 16002 (2016). 10.1088/1478-3975/13/1/016002 PMC4887094 26824302

[b43] Romano D. . Protein interaction switches coordinate Raf-1 and MST2/Hippo signalling. Nat Cell Biol 16, 673–684 (2014). 24929361 10.1038/ncb2986

[b44] Cseh B., Doma E. & Baccarini M. ‘RAF’ neighborhood: Protein–protein interaction in the Raf/Mek/Erk pathway. FEBS Lett. 588, 2398–2406 (2014). 24937142 10.1016/j.febslet.2014.06.025 PMC4099524

[b45] Roskoski R. Jr. RAF protein-serine/threonine kinases: Structure and regulation. Biochem. Biophys. Res. Commun. 399, 313–317 (2010). 20674547 10.1016/j.bbrc.2010.07.092

[b46] Markevich N. I., Hoek J. B. & Kholodenko B. N. Signaling switches and bistability arising from multisite phosphorylation in protein kinase cascades. J. Cell Biol. 164, 353–359 (2004). 14744999 10.1083/jcb.200308060 PMC2172246

[b47] Kiyatkin A. . Scaffolding Protein Grb2-associated Binder 1 Sustains Epidermal Growth Factor-induced Mitogenic and Survival Signaling by Multiple Positive Feedback Loops. J. Biol. Chem. 281, 19925–19938 (2006). 16687399 10.1074/jbc.M600482200 PMC2312093

[b48] Hat B., Puszynski K. & Lipniacki T. Exploring mechanisms of oscillations in p53 and nuclear factor-κB systems. IET Syst. Biol. 3, 342–355 (2009). 21028925 10.1049/iet-syb.2008.0156

[b49] Cheong R., Rhee A., Wang C. J., Nemenman I. & Levchenko A. Information Transduction Capacity of Noisy Biochemical Signaling Networks. Science 334, 354–358 (2011). 21921160 10.1126/science.1204553 PMC3895446

[b50] Selimkhanov J. . Accurate information transmission through dynamic biochemical signaling networks. Science 346, 1370–1373 (2014). 25504722 10.1126/science.1254933 PMC4813785

[b51] Aoki K. . Stochastic ERK Activation Induced by Noise and Cell-to-Cell Propagation Regulates Cell Density-Dependent Proliferation. Mol. Cell 52, 529–540 (2013). 24140422 10.1016/j.molcel.2013.09.015

[b52] Kempiak S. J., Yip S.-C., Backer J. M. & Segall J. E. Local signaling by the EGF receptor. J. Cell Biol. 162, 781–788 (2003). 12952932 10.1083/jcb.200303144 PMC2172819

[b53] Ehrenreiter K. . Raf-1 regulates Rho signaling and cell migration. J. Cell Biol. 168, 955–964 (2005). 15753127 10.1083/jcb.200409162 PMC2171799

[b54] Joslin E. J., Opresko L. K., Wells A., Wiley H. S. & Lauffenburger D. A. EGF-receptor-mediated mammary epithelial cell migration is driven by sustained ERK signaling from autocrine stimulation. J. Cell Sci. 120, 3688–3699 (2007). 17895366 10.1242/jcs.010488

[b55] Kholodenko B. N., Hancock J. F. & Kolch W. Signalling ballet in space and time. Nat. Rev. Mol. Cell Biol. 11, 414–426 (2010). 20495582 10.1038/nrm2901 PMC2977972

[b56] Mori Y., Jilkine A. & Edelstein-Keshet L. Wave-Pinning and Cell Polarity from a Bistable Reaction-Diffusion System. Biophys. J. 94, 3684–3697 (2008). 18212014 10.1529/biophysj.107.120824 PMC2292363

[b57] Hat B., Kazmierczak B. & Lipniacki T. B Cell Activation Triggered by the Formation of the Small Receptor Cluster: A Computational Study. PLoS Comput Biol 7, e1002197 (2011). 21998572 10.1371/journal.pcbi.1002197 PMC3188507

[b58] Nienałtowski K., Włodarczyk M., Lipniacki T. & Komorowski M. Clustering reveals limits of parameter identifiability in multi-parameter models of biochemical dynamics. BMC Syst. Biol. 9, 1–9 (2015). 26415494 10.1186/s12918-015-0205-8 PMC4587803

[b59] Camalet S., Duke T., Jülicher F. & Prost J. Auditory sensitivity provided by self-tuned critical oscillations of hair cells. Proc. Natl. Acad. Sci. USA 97, 3183–3188 (2000). 10737791 10.1073/pnas.97.7.3183 PMC16213

[b60] Faeder J. R., Blinov M. L. & Hlavacek, W. S. In Systems Biology (ed. Maly I. V.) 500, 113–167 (Humana Press, 2009). 10.1007/978-1-59745-525-1_5 19399430

[b61] Hindmarsh A. C. . SUNDIALS: Suite of Nonlinear and Differential/Algebraic Equation Solvers. ACM Trans Math Softw 31, 363–396 (2005).

[b62] Dhooge A., Govaerts W. & Kuznetsov Y. A. MATCONT: A MATLAB Package for Numerical Bifurcation Analysis of ODEs. ACM Trans Math Softw 29, 141–164 (2003).

[b63] Gillespie D. T. Stochastic Simulation of Chemical Kinetics. Annu. Rev. Phys. Chem. 58, 35–55 (2007). 17037977 10.1146/annurev.physchem.58.032806.104637

[b64] Kazmierczak B. & Lipniacki T. Regulation of kinase activity by diffusion and feedback. J. Theor. Biol. 259, 291–296 (2009). 19306885 10.1016/j.jtbi.2009.03.016

[b65] Jaqaman K. . Robust single-particle tracking in live-cell time-lapse sequences. Nat Meth 5, 695–702 (2008). 10.1038/nmeth.1237 PMC2747604 18641657

