# Supplementary material for: Relaxation oscillations and hierarchy of feedbacks in MAPK signaling
Source: Sci Rep. 2017 Jan 3;7:38244. doi: 10.1038/srep38244 (PMC5206726; doi:10.1038/srep38244)
Supplement: Supplementary Information [file srep38244-s1.pdf]

Relaxation oscillations and hierarchy of feedbacks in MAPK signaling

M. KOCHAŃCZYK, P. KOCIENIEWSKI, E. KOZŁOWSKA, J. JARUSZEWICZ-BŁOŃSKA, B. SPARTA, M. PARGETT, J.G. ALBECK, W.S. HLAVACEK, and T. LIPNIACKI

Supporting Information

- **Supplementary Text S1.** Molecular basis of feedbacks relevant to the model.
- **Supplementary Text S2.** Non-dimensional model equations.
- **Supplementary Table S1.** Parameter values.
- **Supplementary Table S2.** Non-dimensional parameter values.
- **Supplementary Table S3.** Parameters of the stochastic model accounting for cell-to-cell variability in EGFR expression dynamics.
- **Supplementary Table S4**. Parameters of the spatial model accounting for extracellular, localized stochastic releases of EGF.
- **Supplementary Figure S1.** Two-dimensional bifurcation diagrams.
- **Supplementary Figure S2.** Effect of multi-site phosphorylation of SOS on oscillatory behavior.
- **Supplementary Figure S3.** Time profiles of active ERK and its two reporters, EKAR3 and ERKTR.
- **Supplementary Figure S4.** Phase space analysis of sensitivity of oscillatory behavior to negative feedback strengths.
- **Supplementary Figure S5.** Influence of extrinsic noise features on the oscillatory behavior.
- **Supplementary Figure S6.** Effect of intrinsic noise on system trajectories.
- **Supplementary Figure S7.** Effect of extrinsic noise on system trajectories.
- **Supplementary Figure S8.** Distributions of active ERK and its frequency spectrum.

Note: Executable model implementations are provided in separate ***Supplementary Data files 1–4****.*
Animation of COMSOL output is provided as a separate ***Video*** *file.*

Supplementary Text S1. Molecular basis of feedbacks relevant to the model.

Positive feedbacks. SOS1, and presumably SOS2 as well, is a target of positive feedback from RAS [1–3]. Somewhat like a classical effector of RAS, such as RAF, SOS1 contains a binding site that preferentially recognizes GTP-loaded (vs. GDP-loaded) RAS. This site, which has been called the allosteric site, is a pocket formed partly by the Ras exchange motif (REM) domain in SOS [1]. It is distinct from the RAS binding site in SOS1's RasGEF domain, which is involved in promoting GDP/GTP exchange [4]. When the allosteric site is occupied by GTP-loaded RAS, SOS1's GEF activity is enhanced relative to the case where the allosteric site is empty or occupied by GDP-loaded RAS [5]. This allosteric enhancement of SOS1's GEF activity represents a positive feedback because GTP-loaded (active) RAS is the product of SOS1's GEF activity. Active RAS and SOS can also participate in a positive feedback mediated by PI3K which promotes membrane recruitment of GAB1, a scaffold for SOS1 and RAS [6]. Another positive feedback arises from the interplay between the CRAF isoform and its substrate MEK. A physical interaction with MEK has been reported to allosterically enhance the kinase activity of CRAF [7]. Furthermore, MEK can increase RAF catalytic activity by contributing to phosphorylation of the N-terminal acidic region of CRAF enabling it to function as an activator in RAF dimers [8]. RAF dimerization may also represent a positive feedback mechanism when RAF dimers stabilize RAS dimers, higher-order oligomers, or RAS nanoclusters [9]. Importantly, all positive feedbacks are of short range: each involves only two proteins, which likely interact with each other at the plasma membrane. Of note, in the ERK/MEK module, a "hidden" positive feedback may arise in the double phosphorylation/dephosphorylation cycle due to distributive phosphorylation and saturability in the dephosphorylation reaction [10].

The vast majority of negative feedbacks in the MAPK/ERK cascade emanate from activated ERK [11], which may be important for ensuring that signals reach ERK before becoming inhibited. These negative feedback loops can be grouped into two categories depending on their position with respect to the positive feedback loops discussed above.

Negative feedback loops downstream of positive feedback loops. There are two well-characterized negative feedback loops that involve ERK-mediated inhibition of MEK and RAF. ERK-mediated phosphorylation of the MEK1 isoform of MEK leads to inhibition of the kinase activities of both MEK1 and MEK2, provided that MEK2 is in complex with MEK1 [18]. As far as RAF is concerned, all its isoforms are hyperphosphorylated by ERK, which has the effect of inhibiting RAF dimerization and disrupting its interaction with RAS, which is an important step in RAF inactivation [19,20].

Negative feedbacks encompassing positive feedbacks. There are two, largely overlapping, feedback loops that involve inhibitory phosphorylation of SOS, mediated either directly by ERK or indirectly by ERK-activated kinase RSK [12]. Phosphorylation of SOS in a C-terminal proline-rich region containing one or more binding sites for Src homology 3 (SH3) domains prevents SOS from interacting with SH3 domain-containing adaptor

proteins, such as GRB2, a binding partner of EGFR [13]. GRB2 also interacts with phosphorylated SHC1 [14], another binding partner of EGFR, and thus GRB2 associates with EGFR both directly and indirectly. Disruption of SOS–GRB2 interaction prevents recruitment of SOS to EGFR via direct and indirect interaction of GRB2 with EGFR. SOS1 contains at least four residues phosphorylated by ERK [15], whereas SOS2 contains only one [16]. This difference suggests that the two isoforms may have different sensitivities to ERK-mediated negative feedbacks. The specificities of ERK and RSK with respect to SOS overlap. Thus, negative feedback to SOS is mediated by a network motif of a feed-forward loop (FFL), a coherent type 3 FFL in the classification scheme of Mangan and Alon [17]. Based on their analysis [17], this FFL can be expected to generate a delay in relief of SOS inhibition upon deactivation of ERK.

Supplementary Text S1 References

1. Margarit SM, Sondermann H, Hall BE, Nagar B, Hoelz A, Pirruccello M, et al. Structural Evidence for Feedback Activation by Ras·GTP of the Ras-Specific Nucleotide Exchange Factor SOS. Cell. 2003;112: 685–695. doi:10.1016/S0092-8674(03)00149-1
2. Boykevisch S, Zhao C, Sondermann H, Philippidou P, Halegoua S, Kuriyan J, et al. Regulation of Ras Signaling Dynamics by Sos-Mediated Positive Feedback. Curr Biol. 2006;16: 2173–2179. doi:10.1016/j.cub.2006.09.033
3. Vo U, Vajpai N, Flavell L, Bobby R, Breeze AL, Embrey KJ, et al. Monitoring Ras Interactions with the Nucleotide Exchange Factor Son of Sevenless (Sos) Using Site-specific NMR Reporter Signals and Intrinsic Fluorescence. J Biol Chem. 2016;291: 1703–1718. doi:10.1074/jbc.M115.691238
4. Boriack-Sjodin PA, Margarit SM, Bar-Sagi D, Kuriyan J. The structural basis of the activation of Ras by Sos. Nature. 1998;394: 337–343. doi:10.1038/28548
5. Iversen L, Tu H-L, Lin W-C, Christensen SM, Abel SM, Iwig J, et al. Ras activation by SOS: Allosteric regulation by altered fluctuation dynamics. Science. 2014;345: 50–54. doi:10.1126/science.1250373
6. Kiyatkin A, Aksamitiene E, Markevich NI, Borisov NM, Hoek JB, Kholodenko BN. Scaffolding Protein Grb2-associated Binder 1 Sustains Epidermal Growth Factor-induced Mitogenic and Survival Signaling by Multiple Positive Feedback Loops. J Biol Chem. 2006;281: 19925–19938. doi:10.1074/jbc.M600482200
7. Leicht DT, Balan V, Zhu J, Kaplun A, Bronisz A, Rana A, et al. MEK-1 activates C-Raf through a Ras-independent mechanism. Biochim Biophys Acta - Mol Cell Res. 2013;1833: 976–986. doi:10.1016/j.bbamcr.2013.01.015
8. Hu J, Stites EC, Yu H, Germino EA, Meharena HS, Stork PJS, et al. Allosteric Activation of Functionally Asymmetric RAF Kinase Dimers. Cell. 2013;154: 1036–1046. doi:10.1016/j.cell.2013.07.046
9. Cho K, Kasai RS, Park J-H, Chigurupati S, Heidorn SJ, van der Hoeven D, et al. Raf Inhibitors Target Ras Spatiotemporal Dynamics. Curr Biol. 2012;22: 945–955. doi:10.1016/j.cub.2012.03.067
10. Markevich NI, Hoek JB, Kholodenko BN. Signaling switches and bistability arising from multisite phosphorylation in protein kinase cascades. J Cell Biol. 2004;164: 353–359. doi:10.1083/jcb.200308060
11. Anderson NG, Maller JL, Tonks NK, Sturgill TW. Requirement for integration of signals from two distinct phosphorylation pathways for activation of MAP kinase. Nature. 1990;343: 651–653. doi:10.1038/343651a0
12. Douville E, Downward J. EGF induced SOS phosphorylation in PC12 cells involves P90 RSK-2. Oncogene. 1997;15: 373–383.

13. Wang Z, Moran MF. Requirement for the Adapter Protein GRB2 in EGF Receptor Endocytosis. Science. 1996;272: 1935–1938. doi:10.1126/science.272.5270.1935

14. Zheng Y, Zhang C, Croucher DR, Soliman MA, St-Denis N, Pasculescu A, et al. Temporal regulation of EGF signalling networks by the scaffold protein Shc1. Nature. 2013;499: 166–171.

15. Kamioka Y, Yasuda S, Fujita Y, Aoki K, Matsuda M. Multiple Decisive Phosphorylation Sites for the Negative Feedback Regulation of SOS1 via ERK. J Biol Chem. 2010;285: 33540–33548. doi:10.1074/jbc.M110.135517

16. Corbalan-Garcia S, Yang SS, Degenhardt KR, Bar-Sagi D. Identification of the mitogen-activated protein kinase phosphorylation sites on human Sos1 that regulate interaction with Grb2. Mol Cell Biol. 1996;16: 5674–5682. doi:10.1128/MCB.16.10.5674

17. Mangan S, Alon U. Structure and function of the feed-forward loop network motif. Proc Natl Acad Sci USA. 2003;100: 11980–11985. doi:10.1073/pnas.2133841100

18. Catalanotti F, Reyes G, Jesenberger V, Galabova-Kovacs G, de Matos Simoes R, Carugo O, et al. A Mek1-Mek2 heterodimer determines the strength and duration of the Erk signal. Nat Struct Mol Biol. 2009;16: 294–303. doi:10.1038/nsmb.1564

19. Kubicek M, Pacher M, Abraham D, Podar K, Eulitz M, Baccarini M. Dephosphorylation of Ser-259 Regulates Raf-1 Membrane Association. J Biol Chem. 2002;277: 7913–7919. doi:10.1074/jbc.M108733200

20. Freeman AK, Ritt DA, Morrison DK. Effects of Raf Dimerization and Its Inhibition on Normal and Disease-Associated Raf Signaling. Mol Cell. 2013;49: 751–758. doi:10.1016/j.molcel.2012.12.018

21. Ciardiello F, McGeady ML, Kim N, Basolo F, Hynes N, Langton BC, et al. Transforming growth factor-alpha expression is enhanced in human mammary epithelial cells transformed by an activated c-Ha-ras protooncogene but not by the c-neu protooncogene, and overexpression of the transforming growth factor-alpha complementary DNA l. Cell Growth Differ. 1990;1: 407–420.

22. Beerli RR, Graus-Porta D, Woods-Cook K, Chen X, Yarden Y, Hynes NE. Neu differentiation factor activation of ErbB-3 and ErbB-4 is cell specific and displays a differential requirement for ErbB-2. Mol Cell Biol. 1995;15: 6496–6505.

Supplementary Text S2. Non-dimensional model equations

Model equations can be non-dimensionalized by expressing reaction rates in units in which dephosphorylation rate of inhibitory phosphosites in SOS, q, equals 1. There are two general types of equations in the model:

- *Association–dissociation processes*:

$$\frac{\mathrm{d}}{\mathrm{d}t}XY = b \times X \times Y - u \times XY$$

  where b is binding rate, u is unbinding rate, and multiplication is denoted by cross (×). Let $\bar{\bar{X}}$ be a total number of X molecules per cell. We can convert amounts X, Y, XY to, respectively, $x = X/\bar{\bar{X}}$, $y = Y/\bar{\bar{X}}$, $xy = XY/\bar{\bar{X}}$ and rates b and u to, respectively, $\beta = b \times \bar{\bar{X}}/q$ and $\upsilon = u/q$. If the above equation for $(\mathrm{d}/\mathrm{d}t)XY$ is satisfied, then the following equation is satisfied as well:

$$\frac{\mathrm{d}}{\mathrm{d}t}xy = \beta \times x \times y - \upsilon \times xy$$

- *Activation–deactivation processes*:

$$\frac{\mathrm{d}}{\mathrm{d}t}S_{\mathrm{act}} = a \times R \times S_{\mathrm{inact}} - d \times S_{\mathrm{act}}$$

  Let $\bar{\bar{R}}$ and $\bar{\bar{S}}$ be total numbers of R ("activator") and S molecules per cell, respectively. We convert molecule numbers $r = R/\bar{\bar{R}}$, $s_{\mathrm{act}} = S_{\mathrm{act}}/\bar{\bar{S}}$, $s_{\mathrm{inact}} = S_{\mathrm{inact}}/\bar{\bar{S}}$, and rates $\alpha = a \times \bar{\bar{R}}/q$, $\delta = d/q$ to obtain a non-dimensionalized equation:

$$\frac{\mathrm{d}}{\mathrm{d}t}s_{\mathrm{act}} = \alpha \times r \times s_{\mathrm{inact}} - \delta \times s_{\mathrm{act}}$$

Therefore, our chemical equations are converted into non-dimensional ones by dividing all reaction rate coefficients by q and by introducing new variables:

- $\mathrm{erk_x} = \mathrm{ERK_x}/\mathrm{ERK_{tot}}$ where $\mathrm{ERK_x}$ stands for any ERK specie,
- $\mathrm{mek_x} = \mathrm{MEK_x}/\ \mathrm{MEK_{tot}}$ where $\mathrm{MEK_x}$ stands for any MEK specie,
- $\mathrm{raf_x} = \mathrm{RAF_x}/\mathrm{RAF_{tot}}$ where $\mathrm{RAF_x}$ stands for any RAF specie,
- $var = VAR/\mathrm{SOS_{tot}}$ where *VAR* stands for any other variable (i.e., EGFR, RAS, SOS, RasGAP, and their phosphoforms and complexes); molecules represented by these variables can form complexes and therefore should be rescaled by a common factor, $\mathrm{SOS_{tot}}$.

Supplementary Table S2 contains numerical values of rescaled molecule numbers and rate parameters.

- $A{\cdot}B$ denotes the amount of complex of A and B (multiplication is written using $\times$).
- Parameters named $f_{\mathrm{A}\mapsto\mathrm{B}}$ denote the strength of a feedback from A to B.

Equations:

$$\begin{aligned}\tfrac{\mathrm{d}}{\mathrm{d}t}egfr_{\mathrm{a}} = {} & \alpha_1 \times EGF \times \big(egfr_{\mathrm{tot}} - (egfr_{\mathrm{a}} + egfr_{\mathrm{i}}{\cdot}sos_{\mathrm{u}} + egfr_{\mathrm{a}}{\cdot}sos_{\mathrm{u}} + egfr_{\mathrm{i}}{\cdot}sos_{\mathrm{u}}{\cdot}ras_{\mathrm{GTP}} \\ & + egfr_{\mathrm{i}}{\cdot}sos_{\mathrm{u}}{\cdot}ras_{\mathrm{GDP}} + egfr_{\mathrm{a}}{\cdot}sos_{\mathrm{u}}{\cdot}ras_{\mathrm{GTP}} + egfr_{\mathrm{a}}{\cdot}sos_{\mathrm{u}}{\cdot}ras_{\mathrm{GDP}})\big) - \delta_1 \times egfr_{\mathrm{a}} \\ & - \beta_1 \times egfr_{\mathrm{a}} \times \big(sos_{\mathrm{tot}} - (sos_{\mathrm{p}} + sos_{\mathrm{pp}} + sos_{\mathrm{ppp}} + sos_{\mathrm{pppp}} + egfr_{\mathrm{i}}{\cdot}sos_{\mathrm{u}} \\ & + egfr_{\mathrm{a}}{\cdot}sos_{\mathrm{u}} + egfr_{\mathrm{i}}{\cdot}sos_{\mathrm{u}}{\cdot}ras_{\mathrm{GDP}} + egfr_{\mathrm{i}}{\cdot}sos_{\mathrm{u}}{\cdot}ras_{\mathrm{GTP}} + egfr_{\mathrm{a}}{\cdot}sos_{\mathrm{u}}{\cdot}ras_{\mathrm{GDP}} \\ & + egfr_{\mathrm{a}}{\cdot}sos_{\mathrm{u}}{\cdot}ras_{\mathrm{GTP}})\big) + \upsilon_{1\mathrm{A}} \times egfr_{\mathrm{a}}{\cdot}sos_{\mathrm{u}}\end{aligned}$$

$$\begin{aligned}\tfrac{\mathrm{d}}{\mathrm{d}t}sos_{\mathrm{p}} = {} & -3 \times \rho_3 \times f_{\mathrm{ERK_{pp}}\mapsto\mathrm{SOS1}} \times erk_{\mathrm{pp}} \times sos_{\mathrm{p}} + 4 \times \rho_3 \times f_{\mathrm{ERK_{pp}}\mapsto\mathrm{SOS1}} \times erk_{\mathrm{pp}} \times \big(sos_{\mathrm{tot}} \\ & - (sos_{\mathrm{p}} + sos_{\mathrm{pp}} + sos_{\mathrm{ppp}} + sos_{\mathrm{pppp}} + egfr_{\mathrm{i}}{\cdot}sos_{\mathrm{u}} + egfr_{\mathrm{a}}{\cdot}sos_{\mathrm{u}} + egfr_{\mathrm{i}}{\cdot}sos_{\mathrm{u}}{\cdot}ras_{\mathrm{GDP}} \\ & + egfr_{\mathrm{i}}{\cdot}sos_{\mathrm{u}}{\cdot}ras_{\mathrm{GTP}} + egfr_{\mathrm{a}}{\cdot}sos_{\mathrm{u}}{\cdot}ras_{\mathrm{GDP}} + egfr_{\mathrm{a}}{\cdot}sos_{\mathrm{u}}{\cdot}ras_{\mathrm{GTP}})\big) - sos_{\mathrm{p}} + 2 \times sos_{\mathrm{pp}}\end{aligned}$$

$$\begin{aligned}\tfrac{\mathrm{d}}{\mathrm{d}t}sos_{\mathrm{pp}} = {} & 3 \times \rho_3 \times f_{\mathrm{ERK_{pp}}\mapsto\mathrm{SOS1}} \times erk_{\mathrm{pp}} \times sos_{\mathrm{p}} + 3 \times sos_{\mathrm{ppp}} - 2 \times sos_{\mathrm{pp}} \\ & - 2 \times \rho_3 \times f_{\mathrm{ERK_{pp}}\mapsto\mathrm{SOS1}} \times erk_{\mathrm{pp}} \times sos_{\mathrm{pp}}\end{aligned}$$

$$\begin{aligned}\tfrac{\mathrm{d}}{\mathrm{d}t}sos_{\mathrm{ppp}} = {} & 2 \times \rho_3 \times f_{\mathrm{ERK_{pp}}\mapsto\mathrm{SOS1}} \times erk_{\mathrm{pp}} \times sos_{\mathrm{pp}} + 4 \times sos_{\mathrm{pppp}} - 3 \times sos_{\mathrm{ppp}} \\ & - \rho_3 \times f_{\mathrm{ERK_{pp}}\mapsto\mathrm{SOS1}} \times erk_{\mathrm{pp}} \times sos_{\mathrm{ppp}}\end{aligned}$$

$$\tfrac{\mathrm{d}}{\mathrm{d}t}sos_{\mathrm{pppp}} = \rho_3 \times f_{\mathrm{ERK_{pp}}\mapsto\mathrm{SOS1}} \times erk_{\mathrm{pp}} \times sos_{\mathrm{ppp}} - 4 \times sos_{\mathrm{pppp}}$$

$$\begin{aligned}\tfrac{\mathrm{d}}{\mathrm{d}t}egfr_{\mathrm{i}}{\cdot}sos_{\mathrm{u}} = {} & \upsilon_{2\mathrm{B}} \times egfr_{\mathrm{i}}{\cdot}sos_{\mathrm{u}}{\cdot}ras_{\mathrm{GDP}} - \alpha_1 \times EGF \times egfr_{\mathrm{i}}{\cdot}sos_{\mathrm{u}} - \upsilon_{1\mathrm{B}} \times egfr_{\mathrm{i}}{\cdot}sos_{\mathrm{u}} \\ & + \delta_1 \times egfr_{\mathrm{a}}{\cdot}sos_{\mathrm{u}} - \beta_{2\mathrm{B}} \times egfr_{\mathrm{i}}{\cdot}sos_{\mathrm{u}} \times \big(ras_{\mathrm{tot}} - (ras_{\mathrm{GTP}} + ras_{\mathrm{GTP}}{\cdot}rasgap \\ & + ras_{\mathrm{GDP}}{\cdot}rasfap + egfr_{\mathrm{i}}{\cdot}sos_{\mathrm{u}}{\cdot}ras_{\mathrm{GDP}} + egfr_{\mathrm{i}}{\cdot}sos_{\mathrm{u}}{\cdot}ras_{\mathrm{GTP}} + egfr_{\mathrm{a}}{\cdot}sos_{\mathrm{u}}{\cdot}ras_{\mathrm{GDP}} \\ & + egfr_{\mathrm{a}}{\cdot}sos_{\mathrm{u}}{\cdot}ras_{\mathrm{GTP}})\big) + \upsilon_{2\mathrm{A}} \times egfr_{\mathrm{i}}{\cdot}sos_{\mathrm{u}}{\cdot}ras_{\mathrm{GTP}} - \beta_{2\mathrm{A}} \times egfr_{\mathrm{i}}{\cdot}sos_{\mathrm{u}} \times ras_{\mathrm{GTP}}\end{aligned}$$

$$\begin{aligned}\tfrac{\mathrm{d}}{\mathrm{d}t}egfr_{\mathrm{a}}{\cdot}sos_{\mathrm{u}} = {} & \beta_1 \times egfr_{\mathrm{a}} \times (sos_{\mathrm{tot}} - (sos_{\mathrm{p}} + sos_{\mathrm{pp}} + sos_{\mathrm{ppp}} + sos_{\mathrm{pppp}} \\ & + egfr_{\mathrm{i}}{\cdot}sos_{\mathrm{u}} + egfr_{\mathrm{a}}{\cdot}sos_{\mathrm{u}} + egfr_{\mathrm{i}}{\cdot}sos_{\mathrm{u}}{\cdot}ras_{\mathrm{GDP}} \\ & + egfr_{\mathrm{i}}{\cdot}sos_{\mathrm{u}}{\cdot}ras_{\mathrm{GTP}} + egfr_{\mathrm{a}}{\cdot}sos_{\mathrm{u}}{\cdot}ras_{\mathrm{GDP}} + egfr_{\mathrm{a}}{\cdot}sos_{\mathrm{u}}{\cdot}ras_{\mathrm{GTP}})\big) \\ & - \beta_{2\mathrm{B}} \times egfr_{\mathrm{a}}{\cdot}sos_{\mathrm{u}} \times \big(ras_{\mathrm{tot}} - (ras_{\mathrm{GTP}} + ras_{\mathrm{GTP}}{\cdot}rassgap + ras_{\mathrm{GDP}}{\cdot}rasgap \\ & + egfr_{\mathrm{i}}{\cdot}sos_{\mathrm{u}}{\cdot}ras_{\mathrm{GDP}} + egfr_{\mathrm{i}}{\cdot}sos_{\mathrm{u}}{\cdot}ras_{\mathrm{GTP}} + egfr_{\mathrm{a}}{\cdot}sos_{\mathrm{u}}{\cdot}ras_{\mathrm{GDP}} \\ & + egfr_{\mathrm{a}}{\cdot}sos_{\mathrm{u}}{\cdot}ras_{\mathrm{GTP}})\big) - \upsilon_{1\mathrm{A}} \times egfr_{\mathrm{a}}{\cdot}sos_{\mathrm{u}} + \upsilon_{2\mathrm{A}} \times egfr_{\mathrm{a}}{\cdot}sos_{\mathrm{u}}{\cdot}ras_{\mathrm{GTP}} \\ & + \upsilon_{2\mathrm{B}} \times egfr_{\mathrm{a}}{\cdot}sos_{\mathrm{u}}{\cdot}ras_{\mathrm{GDP}} + \alpha_1 \times EGF \times egfr_{\mathrm{i}}{\cdot}sos_{\mathrm{u}} \\ & - \beta_{2\mathrm{A}} \times egfr_{\mathrm{a}}{\cdot}sos_{\mathrm{u}} \times ras_{\mathrm{GTP}} - \delta_1 \times egfr_{\mathrm{a}}{\cdot}sos_{\mathrm{u}}\end{aligned}$$

$$
\begin{aligned}
\tfrac{\mathrm{d}}{\mathrm{d}t} ras_{\mathrm{GTP}} = {} & \kappa_{2\mathrm{B}} \times egfr_{\mathrm{i}}{\cdot}sos_{\mathrm{u}}{\cdot}ras_{\mathrm{GDP}} \times \big(ras_{\mathrm{tot}} - (ras_{\mathrm{GTP}} + ras_{\mathrm{GTP}}{\cdot}rasgap + ras_{\mathrm{GDP}}{\cdot}rasgap \\
& + egfr_{\mathrm{i}}{\cdot}sos_{\mathrm{u}}{\cdot}ras_{\mathrm{GDP}} + egfr_{\mathrm{i}}{\cdot}sos_{\mathrm{u}}{\cdot}ras_{\mathrm{GTP}} + egfr_{\mathrm{a}}{\cdot}sos_{\mathrm{u}}{\cdot}ras_{\mathrm{GDP}} \\
& + egfr_{\mathrm{a}}{\cdot}sos_{\mathrm{u}}{\cdot}ras_{\mathrm{GTP}})\big) + \upsilon_{2\mathrm{A}} \times egfr_{\mathrm{i}}{\cdot}sos_{\mathrm{u}}{\cdot}ras_{\mathrm{GTP}} - \beta_{2\mathrm{A}} \times egfr_{\mathrm{a}}{\cdot}sos_{\mathrm{u}} \times ras_{\mathrm{GTP}} \\
& + \kappa_{2\mathrm{B}} \times egfr_{\mathrm{a}}{\cdot}sos_{\mathrm{u}}{\cdot}ras_{\mathrm{GDP}} \times \big(ras_{\mathrm{tot}} - (ras_{\mathrm{GTP}} + ras_{\mathrm{GTP}}{\cdot}rasgap + ras_{\mathrm{GDP}}{\cdot}rasgap \\
& + egfr_{\mathrm{i}}{\cdot}sos_{\mathrm{u}}{\cdot}ras_{\mathrm{GDP}} + egfr_{\mathrm{i}}{\cdot}sos_{\mathrm{u}}{\cdot}ras_{\mathrm{GTP}} + egfr_{\mathrm{a}}{\cdot}sos_{\mathrm{u}}{\cdot}rasS_{\mathrm{GDP}} \\
& + egfr_{\mathrm{a}}{\cdot}sos_{\mathrm{u}}{\cdot}ras_{\mathrm{GTP}})\big) + \kappa_{2\mathrm{A}} \times egfr_{\mathrm{i}}{\cdot}sos_{\mathrm{u}}{\cdot}ras_{\mathrm{GTP}} \times \big(ras_{\mathrm{tot}} - (ras_{\mathrm{GTP}} \\
& + ras_{\mathrm{GTP}}{\cdot}rasgap + ras_{\mathrm{GDP}}{\cdot}rasgap + egfr_{\mathrm{i}}{\cdot}sos_{\mathrm{u}}{\cdot}ras_{\mathrm{GDP}} + egfr_{\mathrm{i}}{\cdot}sos_{\mathrm{u}}{\cdot}ras_{\mathrm{GTP}} \\
& + egfr_{\mathrm{a}}{\cdot}sos_{\mathrm{u}}{\cdot}ras_{\mathrm{GDP}} + egfr_{\mathrm{a}}{\cdot}sos_{\mathrm{u}}{\cdot}ras_{\mathrm{GTP}})\big) + \upsilon_{2\mathrm{A}} \times egfr_{\mathrm{a}}{\cdot}sos_{\mathrm{u}}{\cdot}ras_{\mathrm{GTP}} \\
& - \beta_{2\mathrm{A}} \times egfr_{\mathrm{i}}{\cdot}sos_{\mathrm{u}} \times ras_{\mathrm{GTP}}) + \kappa_{2\mathrm{A}} \times egfr_{\mathrm{a}}{\cdot}sos_{\mathrm{u}}{\cdot}ras_{\mathrm{GTP}} \times \big(ras_{\mathrm{tot}} - (ras_{\mathrm{GTP}} \\
& + ras_{\mathrm{GTP}}{\cdot}rasgap + ras_{\mathrm{GDP}}{\cdot}rasfap + egfr_{\mathrm{i}}{\cdot}sos_{\mathrm{u}}{\cdot}ras_{\mathrm{GDP}} + egfr_{\mathrm{i}}{\cdot}sos_{\mathrm{u}}{\cdot}ras_{\mathrm{GTP}} \\
& + egfr_{\mathrm{a}}{\cdot}sos_{\mathrm{u}}{\cdot}ras_{\mathrm{GDP}} + egfr_{\mathrm{a}}{\cdot}sos_{\mathrm{u}}{\cdot}ras_{\mathrm{GTP}})\big) - \beta_3 \times \big(rasgap_{\mathrm{tot}} - (ras_{\mathrm{GTP}}{\cdot}rasgap \\
& + ras_{\mathrm{GDP}}{\cdot}rasgap)\big) \times ras_{\mathrm{GTP}}
\end{aligned}
$$

$$
\begin{aligned}
\tfrac{\mathrm{d}}{\mathrm{d}t} egfr_{\mathrm{i}}{\cdot}sos_{\mathrm{u}}{\cdot}ras_{\mathrm{GDP}} = {} & -\upsilon_{2\mathrm{B}} \times egfr_{\mathrm{i}}{\cdot}sos_{\mathrm{u}}{\cdot}ras_{\mathrm{GDP}} + \delta_1 \times egfr_{\mathrm{a}}{\cdot}sos_{\mathrm{u}}{\cdot}ras_{\mathrm{GDP}} \\
& - \alpha_1 \times EGF \times egfr_{\mathrm{i}}{\cdot}sos_{\mathrm{u}}{\cdot}ras_{\mathrm{GDP}} + \beta_{2\mathrm{B}} \times egfr_{\mathrm{i}}{\cdot}sos_{\mathrm{u}} \\
& \times \big(ras_{\mathrm{tot}} - (ras_{\mathrm{GTP}} + ras_{\mathrm{GTP}}{\cdot}rasgap + ras_{\mathrm{GDP}}{\cdot}rasgap + egfr_{\mathrm{i}}{\cdot}sos_{\mathrm{u}}{\cdot}ras_{\mathrm{GDP}} \\
& + egfr_{\mathrm{i}}{\cdot}sos_{\mathrm{u}}{\cdot}ras_{\mathrm{GTP}} + egfr_{\mathrm{a}}{\cdot}sos_{\mathrm{u}}{\cdot}ras_{\mathrm{GDP}} + egfr_{\mathrm{a}}{\cdot}sos_{\mathrm{u}}{\cdot}ras_{\mathrm{GTP}})\big)
\end{aligned}
$$

$$
\begin{aligned}
\tfrac{\mathrm{d}}{\mathrm{d}t} egfr_{\mathrm{i}}{\cdot}sos_{\mathrm{u}}{\cdot}ras_{\mathrm{GTP}} = {} & \beta_{2\mathrm{A}} \times egfr_{\mathrm{i}}{\cdot}sos_{\mathrm{u}} \times ras_{\mathrm{GTP}} - \alpha_1 \times EGF \times egfr_{\mathrm{i}}{\cdot}sos_{\mathrm{u}}{\cdot}ras_{\mathrm{GTP}} \\
& - \upsilon_{2\mathrm{A}} \times egfr_{\mathrm{i}}{\cdot}sos_{\mathrm{u}}{\cdot}ras_{\mathrm{GTP}} + \delta_1 \times egfr_{\mathrm{a}}{\cdot}sos_{\mathrm{u}}{\cdot}ras_{\mathrm{GTP}}
\end{aligned}
$$

$$
\begin{aligned}
\tfrac{\mathrm{d}}{\mathrm{d}t} egfr_{\mathrm{a}}{\cdot}sos_{\mathrm{u}}{\cdot}ras_{\mathrm{GDP}} = {} & \beta_{2\mathrm{B}} \times egfr_{\mathrm{a}}{\cdot}sos_{\mathrm{u}} \times \big(ras_{\mathrm{tot}} - (ras_{\mathrm{GTP}} + ras_{\mathrm{GTP}}{\cdot}rasgap + ras_{\mathrm{GDP}}{\cdot}rasgap \\
& + egfr_{\mathrm{i}}{\cdot}sos_{\mathrm{u}}{\cdot}ras_{\mathrm{GDP}} + egfr_{\mathrm{i}}{\cdot}sos_{\mathrm{u}}{\cdot}ras_{\mathrm{GTP}} + egfr_{\mathrm{a}}{\cdot}sos_{\mathrm{u}}{\cdot}ras_{\mathrm{GDP}} \\
& + egfr_{\mathrm{a}}{\cdot}sos_{\mathrm{u}}{\cdot}ras_{\mathrm{GTP}})\big) - \delta_1 \times egfr_{\mathrm{a}}{\cdot}sos_{\mathrm{u}}{\cdot}ras_{\mathrm{GDP}} \\
& + \alpha_1 \times EGF \times egfr_{\mathrm{i}}{\cdot}sos_{\mathrm{u}}{\cdot}ras_{\mathrm{GDP}} - \upsilon_{2\mathrm{B}} \times egfr_{\mathrm{a}}{\cdot}sos_{\mathrm{u}}{\cdot}ras_{\mathrm{GDP}}
\end{aligned}
$$

$$
\begin{aligned}
\tfrac{\mathrm{d}}{\mathrm{d}t} egfr_{\mathrm{a}}{\cdot}sos_{\mathrm{u}}{\cdot}ras_{\mathrm{GTP}} = {} & \alpha_1 \times EGF \times egfr_{\mathrm{i}}{\cdot}sos_{\mathrm{u}}{\cdot}ras_{\mathrm{GTP}} + \beta_{2\mathrm{A}} \times egfr_{\mathrm{a}}{\cdot}sos_{\mathrm{u}} \times ras_{\mathrm{GTP}} \\
& - \upsilon_{2\mathrm{A}} \times egfr_{\mathrm{a}}{\cdot}sos_{\mathrm{u}}{\cdot}ras_{\mathrm{GTP}} - \delta_1 \times egfr_{\mathrm{a}}{\cdot}sos_{\mathrm{u}} \times ras_{\mathrm{GTP}}
\end{aligned}
$$

$$
\tfrac{\mathrm{d}}{\mathrm{d}t} ras_{\mathrm{GDP}}{\cdot}rasgap = -\upsilon_3 \times ras_{\mathrm{GDP}}{\cdot}rasgap + \kappa_3 \times ras_{\mathrm{GTP}}{\cdot}rasgap
$$

$$
\tfrac{\mathrm{d}}{\mathrm{d}t} ras_{\mathrm{GTP}}{\cdot}rasgap = -\kappa_3 \times ras_{\mathrm{GTP}}{\cdot}rasgap + \beta_3 \times \big(rasgap_{\mathrm{tot}} - (ras_{\mathrm{GTP}}{\cdot}rasgap + ras_{\mathrm{GDP}}{\cdot}rasgap)\big) \times ras_{\mathrm{GTP}}
$$

$$\frac{d}{dt} raf_a = -\rho_6 \times f_{ERK_{pp} \mapsto RAF} \times erk_{pp} \times raf_a - \delta_2 \times raf_a + \alpha_2 \times ras_{GTP} \times \left(raf_{tot} - (raf_a + raf_p)\right)$$

$$\frac{d}{dt} raf_p = \rho_6 \times f_{ERK_{pp} \mapsto RAF} \times erk_{pp} \times raf_a + \rho_6 \times f_{ERK_{pp} \mapsto RAF} \times erk_{pp} \times \left(raf_{tot} - (raf_a + raf_p)\right) - raf_p$$

$$\begin{aligned}\frac{d}{dt} mek_{u,T292p} = {} & -mek_{u,T292p} + \rho_4 \times f_{ERK_{pp} \mapsto MEK1} \times erk_{pp} \times \big(mek_{tot} - (mek_{u,T292p} + mek_{p,T292u} \\ & + mek_{p,T292p} + mek_{pp,T292u} + mek_{pp,T292p})\big) + \sigma_1 \times mek_{p,T292p} + \sigma_5 \times mek_{p,T292p} \\ & - 2 \times \rho_1 \times mek_{p,T292p} \times raf_a\end{aligned}$$

$$\begin{aligned}\frac{d}{dt} mek_{p,T292u} = {} & mek_{p,T292p} - \sigma_1 \times mek_{p,T292u} + 2 \times q_1 \times mek_{pp,T292u} - \sigma_1 \times mek_{p,T292u} \times raf_a \\ & + 2 \times \rho_1 \times \big(mek_{tot} - (mek_{u,T292p} + mek_{p,T292u} + mek_{p,T292p} + mek_{pp,T292u} \\ & + mek_{pp,T292p})\big) \times raf_a - \rho_4 \times f_{ERK_{pp} \mapsto MEK1} \times erk_{pp} \times mek_{p,T292u}\end{aligned}$$

$$\begin{aligned}\frac{d}{dt} mek_{p,T292p} = {} & \rho_4 \times f_{ERK_{pp} \mapsto MEK1} \times erk_{pp} \times mek_{p,T292u} - \sigma_1 \times mek_{p,T292p} - \rho_1 \times mek_{p,T292p} \times raf_a \\ & - mek_{p,T292p} - \sigma_5 \times mek_{p,T292p} + 2 \times \sigma_5 \times mek_{pp,T292p} + 2 \times \sigma_1 \times mek_{pp,T292p} \\ & + 2 \times \rho_1 \times mek_{u,T292p} \times raf_a\end{aligned}$$

$$\begin{aligned}\frac{d}{dt} mek_{pp,T292u} = {} & -\rho_4 \times f_{ERK_{pp} \mapsto MEK1} \times erk_{pp} \times mek_{pp,T292u} - 2 \times \sigma_1 \times mek_{pp,T292u} + \rho_1 \times mek_{p,T292u} \times raf_a \\ & + mek_{pp,T292p}\end{aligned}$$

$$\begin{aligned}\frac{d}{dt} mek_{pp,T292p} = {} & \rho_1 \times mek_{p,T292p} \times raf_a + \rho_4 \times f_{ERK_{pp} \mapsto MEK1} \times erk_{pp} \times mek_{pp,T292u} - mek_{pp,T292p} \\ & - 2 \times \sigma_1 \times mek_{pp,T292p} - 2 \times \sigma_5 \times mek_{pp,T292p}\end{aligned}$$

$$\begin{aligned}\frac{d}{dt} erk_p = {} & 2 \times \sigma_2 \times erk_{pp} + 2 \times \rho_2 \times mek_{pp,T292u} \times \big(erk_{tot} - (erk_p + erk_{pp})\big) - \sigma_2 \times erk_p \\ & - \rho_2 \times mek_{pp,T292u} \times erk_p + 2 \times \rho_2 \times mek_{pp,T292p} \times \big(erk_{tot} - (erk_p + erk_{pp})\big) \\ & - \rho_2 \times mek_{pp,T292p} \times erk_p\end{aligned}$$

$$\frac{d}{dt} erk_{pp} = \rho_2 \times mek_{pp,T292p} \times erk_p - 2 \times \sigma_2 \times erk_{pp} + \rho_2 \times mek_{pp,T292u} \times erk_p$$

Supplementary Tables

Supplementary Table S1. Parameter values.

**Parameter**	**Description (see also Fig. 2 in the main text)**
Concentrations (protein copy number per cell)	
$EGFR_{tot} = 3\times10^5$	Total number of EGFR molecules (as reported for MCF10A cells [21,22])
$RAS_{tot} = 6\times10^4$	Total number of RAS molecules
$SOS_{tot} = 10^5$	Total number of SOS molecules
$RasGAP_{tot} = 6\times10^3$	Total number of RasGAP molecules
$RAF_{tot} = 5\times10^5$	Total number of RAF molecules
$MEK_{tot} = 2\times10^5$	Total number of MEK molecules
$ERK_{tot} = 3\times10^6$	Total number of ERK molecules
Rate constant for fast processes (s^{-1})	
$k_{fast} = 100$	As a simplification, three (pseudo) first-order processes considered in the model are assumed to be fast.
Rate constants for activation processes [(molecules/cell)$^{-1}$ s^{-1} except (pg/ml)$^{-1}$ s^{-1} for a_1]	
$a_1 = 5\times10^{-5}$	EGF-mediated activation of EGFR
$a_2 = 10^{-7}$	Activation of RAF by RAS-GTP
$b_1 = 10^{-5}$	Binding of EGF-activated EGFR and dephosphorylated SOS
$p_1 = 10^{-7}$	Phosphorylation of MEK's activation sites by RAF
$p_2 = 3\times10^{-6}$	Phosphorylation of ERK's activation sites by MEK
Rate constants for deactivation processes (s^{-1})	
$d_1 = d = 0.01$	Deactivation of EGFR
$d_2 = d$	Deactivation of RAF (spontaneous)
$u_{1A} = d$	Dissociation of active EGFR–SOS complex

$q_1 = d$	Dephosphorylation of MEK's activation sites
$q_2 = d$	Dephosphorylation of ERK's activation sites
Rate constants for SOS association reactions [(molecules/cell)$^{-1}$ s^{-1}] *and dissociation reactions* (s^{-1})	
$b_{2A} = 10^{-6}$	Association of SOS and RAS-GTP
$b_{2B} = 0.1 \times b_{2A}$	Association of SOS and RAS-GDP
$u_{1B} = k_{fast}$	Dissociation of inactive EGFR–SOS complex
$u_{2A} = u_{2B} = 1$	Dissociation of SOS–RAS-GTP complex and SOS–RAS-GDP complex
Rate constants associated with SOS-to-RAS positive feedback [(molecules/cell)$^{-1}$ s^{-1} except s^{-1} for k_3 and u_3]	
$k_{2A} = 10^{-4}$	SOS's nucleotide-exchange activity when RAS-GTP is bound to its REM domain
$k_{2B} = 0.1 \times k_{2A}$	SOS's nucleotide-exchange activity when RAS-GDP is bound to its REM domain
$k_{2C} = 0$	SOS's nucleotide-exchange activity when no RAS is bound to its REM domain
$b_3 = 10^{-5}$	Association of RasGAP and RAS-GTP
$k_3 = k_{fast}$	RasGAP-facilitated RAS-GTP→RAS-GDP hydrolysis
$u_3 = 0.01$	Dissociation of RasGAP from RAS-GDP after hydrolysis of GTP
Rate constants associated with ERK-mediated negative feedbacks [(molecules/cell)$^{-1}$ s^{-1}]	
$p_3 = 3{\times}10^{-9}$	Phosphorylation of unbound SOS by active ERK
$p_4 = p_6 = p = 6{\times}10^{-10}$	Phosphorylation of MEK (T292 in MEK1) and RAF by active ERK
Rate constants for (spontaneous) dephosphorylation reactions (s^{-1})	
$q_3 = q_4 = q_6 = q = 3{\times}10^{-4}$	Dephosphorylation of inhibitory phosphosites in SOS, MEK (T292 in MEK1), RAF
$q_5 = k_{fast}$	Dephosphorylation of MEK's activation sites when T292 (in MEK1) is phosphorylated

ERK activity reporters – concentrations (protein copy number per cell)	
$\mathrm{EKAR3_{tot}} = \mathrm{ERKTR_{tot}} = 10^6$	Total number of EKAR3 and ERKTR molecules
ERK activity reporters – rate constants for activation reactions [(molecules/cell)$^{-1}$ s^{-1}]	
$a_\mathrm{EKAR3} = 3\times10^{-9}$	EKAR3 activation rate by active ERK
$a_\mathrm{ERKTR} = 10^{-9}$	ERKTR activation rate by active ERK
ERK activity reporters – rate constants for deactivation reactions (s^{-1})	
$d_\mathrm{EKAR3} = 10^{-3}$	EKAR3 deactivation rate
$d_\mathrm{ERKTR} = 2\times10^{-3}$	ERKTR deactivation rate

Supplementary Table S2. Non-dimensional parameter values.

**Non-dimensional parameter**	**Description**
Concentrations	
$egfr_{\mathrm{tot}} = 3$	Total number of EGFR molecules
$ras_{\mathrm{tot}} = 0.6$	Total number of RAS molecules
$sos_{\mathrm{tot}} = 1$	Total number of SOS molecules
$rasgap_{\mathrm{tot}} = 0.06$	Total number of RasGAP molecules
$raf_{\mathrm{tot}} = 1$	Total number of RAF molecules
$mek_{\mathrm{tot}} = 1$	Total number of MEK molecules
$erk_{\mathrm{tot}} = 1$	Total number of ERK molecules
Rate constant for fast processes	
$\kappa_{\mathrm{fast}} = \frac{1}{3} \times 10^6$	As a simplification, three (pseudo) first-order processes considered in the model are assumed to be fast.
Rate constants for activation processes	
$\alpha_1 = \frac{1}{6}$	EGF-mediated activation of EGFR
$\alpha_2 = \frac{1}{3} \times 10^2$	Activation of RAF by RAS-GTP
$\beta_1 = \frac{1}{3} \times 10^4$	Binding of EGF-activated EGFR and dephosphorylated SOS
$\rho_1 = 166\,\frac{2}{3}$	Phosphorylation of MEK's activation sites by RAF
$\rho_2 = 2 \times 10^3$	Phosphorylation of ERK's activation sites by MEK
Rate constants for deactivation processes	
$\delta_1 = \delta = \frac{1}{3} \times 10^2$	Deactivation of EGFR
$\delta_2 = \delta$	Deactivation of RAF (spontaneous)
$\upsilon_{1A} = \delta$	Dissociation of active EGFR–SOS complex

$\sigma_1 = \delta$	Dephosphorylation of MEK's activation sites
$\sigma_2 = \delta$	Dephosphorylation of ERK's activation sites
Rate constants for SOS association reactions	
$\beta_{2A} = \frac{1}{3} \times 10^3$	Association of SOS and RAS-GTP
$\beta_{2B} = 0.1 \times \beta_{2A}$	Association of SOS and RAS-GDP
$\upsilon_{1B} = \kappa_{\text{fast}}$	Dissociation of inactive EGFR–SOS complex
$\upsilon_{2A} = \upsilon_{2B} = \frac{1}{3} \times 10^4$	Dissociation of SOS–RAS-GTP complex and SOS–RAS-GDP complex
Rate constants associated with SOS-to-RAS positive feedback	
$\kappa_{2A} = \frac{1}{3} \times 10^5$	SOS's nucleotide-exchange activity when RAS-GTP is bound to its REM domain
$\kappa_{2B} = 0.1 \times \kappa_{2A}$	SOS's nucleotide-exchange activity when RAS-GDP is bound to its REM domain
$\kappa_{2C} = 0$	SOS's nucleotide-exchange activity when no RAS is bound to its REM domain
$\beta_3 = \frac{1}{3} \times 10^4$	Association of RasGAP and RAS-GTP
$\kappa_3 = \kappa_{\text{fast}}$	RasGAP-facilitated RAS-GTP→RAS-GDP hydrolysis
$\upsilon_3 = \frac{1}{3} \times 10^2$	Dissociation of RasGAP from RAS-GDP after hydrolysis of GTP
Rate constants associated with ERK-mediated negative feedbacks	
$\rho_3 = 30$	Phosphorylation of unbound SOS by active ERK
$\rho_4 = \rho_6 = \rho = 6$	Phosphorylation of MEK (T292 in MEK1) and RAF by active ERK
Rate constants for (spontaneous) dephosphorylation reactions	
1 (time scale)	Dephosphorylation of inhibitory phosphosites in SOS, MEK (T292 in MEK1), RAF
$\sigma_5 = \kappa_{\text{fast}}$	Dephosphorylation of MEK's activation sites when T292 (in MEK1) is phosphorylated

Supplementary Table S3. Parameters of the stochastic model accounting for cell-to-cell variability in EGFR expression dynamics. These parameters, together with parameters in Supplementary Table S1, were used to perform simulations shown in Fig. 8 in the main text.

**Parameter**	**Description**
$\mu_{\mathrm{EGFR}} = -0.03125$, $\sigma_{\mathrm{EGFR}} = 0.25$<br><br>$A_{\mathrm{burst}} = 60\ \mathrm{s}^{-1}$	Intensity of EGFR production within each burst in a cell is computed as $A_{\mathrm{burst}} \times \alpha_{\mathrm{cell}}$, where α_{cell} has the log-normal distribution $\ln N(\mu_{\mathrm{EGFR}}, \sigma_{\mathrm{EGFR}})$ with mean of 1.0 and variance of about 0.0645.
$\lambda_{\mathrm{interburst}} = 1/6\ \mathrm{h}^{-1}$	Waiting times between EGFR bursts are distributed exponentially with a mean waiting time of $\lambda_{\mathrm{interburst}}^{-1}$.
$\lambda_{\mathrm{burst}} = 1\ \mathrm{h}^{-1}$	Durations of EGFR bursts are distributed exponentially with mean burst duration of $\lambda_{\mathrm{burst}}^{-1}$.
$\tau_{\mathrm{EGFR}} = 12$ h	EGFR half-life.

Supplementary Table S4. Parameters of the spatial model accounting for extracellular, localized stochastic releases of EGF. These parameters, together with parameters in Supplementary Table S1, were used to perform simulations shown in Fig. 10 and Supplementary Video 1.

Parameter	Description
$V_{\mathrm{cell}} = 2000\ \mu\mathrm{m}^3$	Volume and radius of the (spherical) cell
$R_{cell} \approx 7.8\ \mu\mathrm{m}$	
$D_{\mathrm{mem}} = 10^{-2}\ \mu\mathrm{m}^2/\mathrm{s}$	Diffusion coefficient of membrane proteins: EGFR, SOS, RAS, and RasGAP, and their complexes
$D_{\mathrm{cyt}} = 10 \times D_{\mathrm{mem}} = 10^{-1}\ \mu\mathrm{m}^2/\mathrm{s}$	Diffusion coefficient of cytosolic proteins: RAF, MEK, ERK
$R_{\mathrm{EGF\_shell\_in}} = R_{\mathrm{cell}} \approx 7.8\ \mu\mathrm{m}$	Inner and outer radii: point sources of EGF randomly appear uniformly within a concentric shell around a cell
$R_{\mathrm{EGF\_shell\_out}} = 10 \times R_{\mathrm{cell}} \approx 78\ \mu\mathrm{m}$	
$\mu_{\mathrm{EGF}} = -1.25$, $\sigma_{\mathrm{EGF}} = 0.5$	EGF source intensity, A_{EGF}, is drawn from a log-normal distribution $\ln N(\mu_{\mathrm{EGF}}, \sigma_{\mathrm{EGF}})$ with mean 3.25×10^{-1} and variance 2.99×10^{-2}
$D_{\mathrm{EGF}} = 10 \times D_{\mathrm{cyt}} = 1\ \mu\mathrm{m}^2/\mathrm{s}$	Diffusion coefficient of EGF
$\gamma_{\mathrm{EGF}} = 8.33 \times 10^{-4}\ \mathrm{s}^{-1}$	EGF degradation rate constant
$\lambda_{\mathrm{EGF\_source}} = 8.33 \times 10^{-4}\ \mathrm{s}^{-1}$	EGF-releasing point source durations are drawn from an exponential distribution with mean $\lambda_{\mathrm{EGF\_source}}^{-1} = 20$ min
$f_{\mathrm{EGF\_sources}} = 1.11 \times 10^{-3}\ \mathrm{s}^{-1}$	EGF sources appear at random time points during the simulation, on average every 15 min

Supplementary Figures

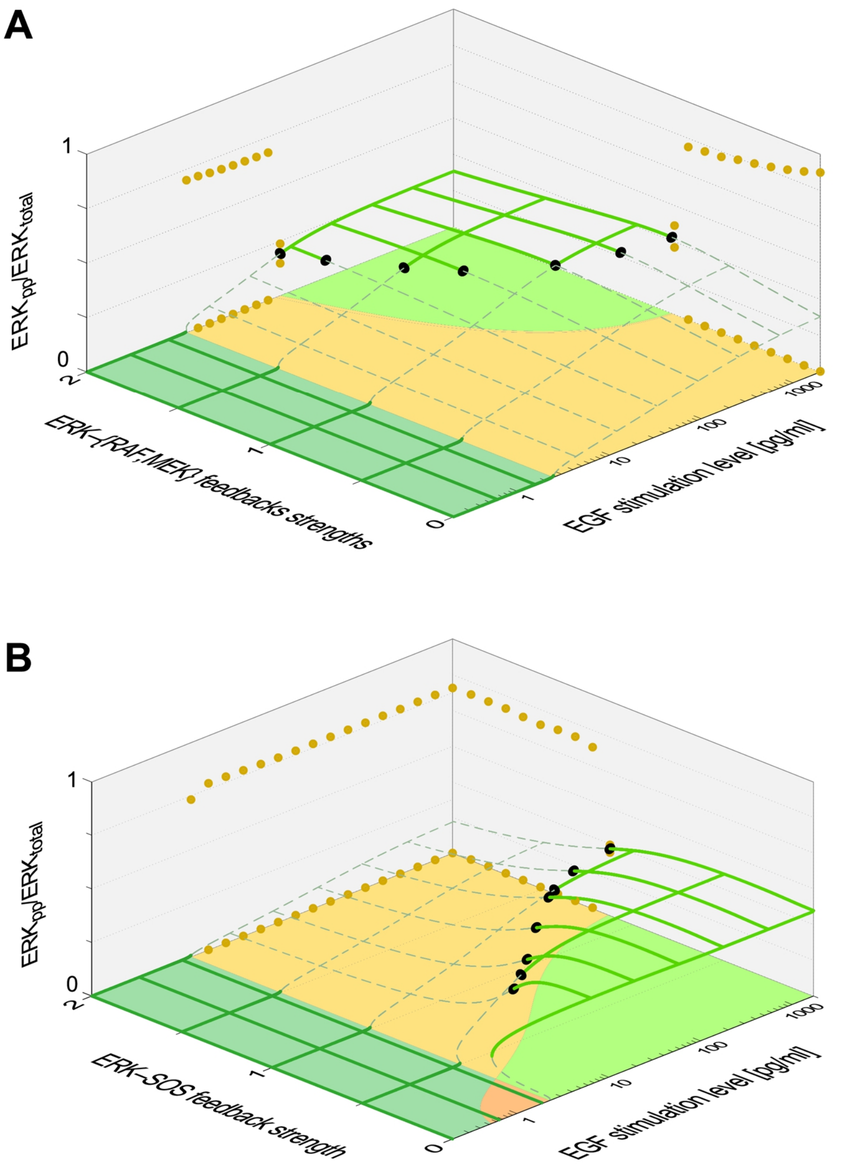

Supplementary Figure S1. Two-dimensional bifurcation diagrams. These diagrams complement Figs. 3A (**A**) and 3B (**B**) in the main text. In each panel, the *x*- and *y*-axes indicate the values of bifurcation parameters, as in Figs. 3A and 3B. The *z*-axis indicates the values of recurrent solutions (i.e., steady states and limit cycles) for activated ERK. Yellow dots mark the envelopes (i.e., the upper and lower bounds) of limit cycles. As in Fig. 5, black dots indicate supercritical Hopf bifurcation points.

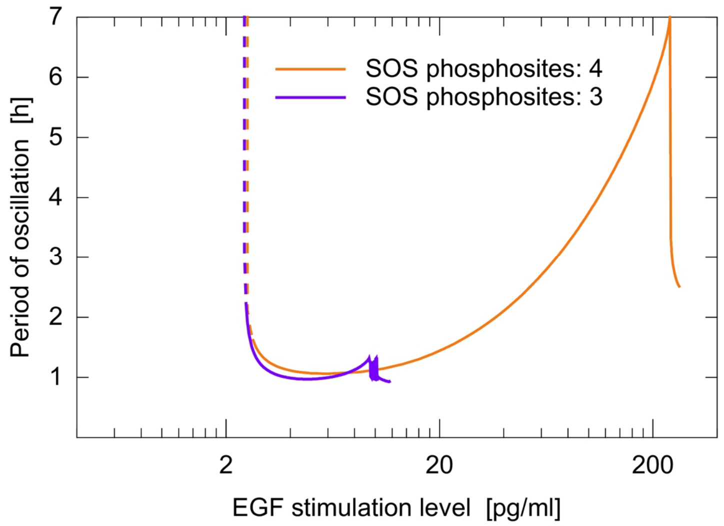

Supplementary Figure S2. Effect of multi-site phosphorylation of SOS on oscillatory behavior. Ablation of a single ERK substrate in SOS decreases the range of EGF stimulation for which oscillations in ERK activity are observed. When SOS contains only one or two ERK substrates, the oscillatory regime vanishes.

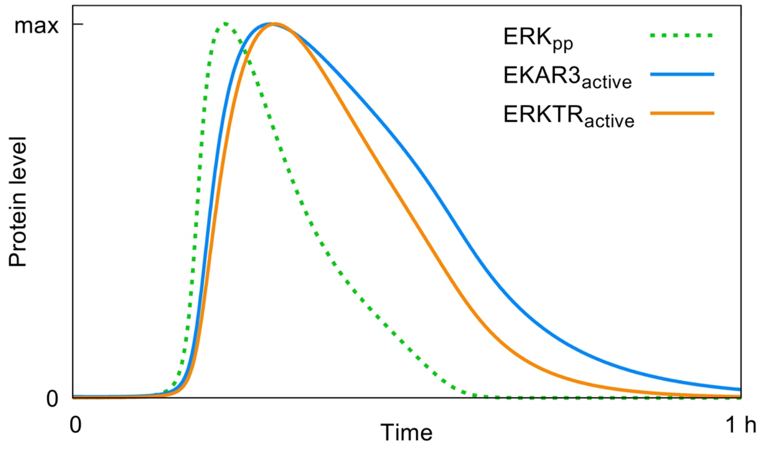

Supplementary Figure S3. Time profiles of active ERK (ERK_{pp}) and its two reporters, EKAR3 and ERKTR. Trajectories were obtained for an EGF dose of 5 pg/ml. Activation and deactivation rates of both reporters are given in Table 1 in the main text.

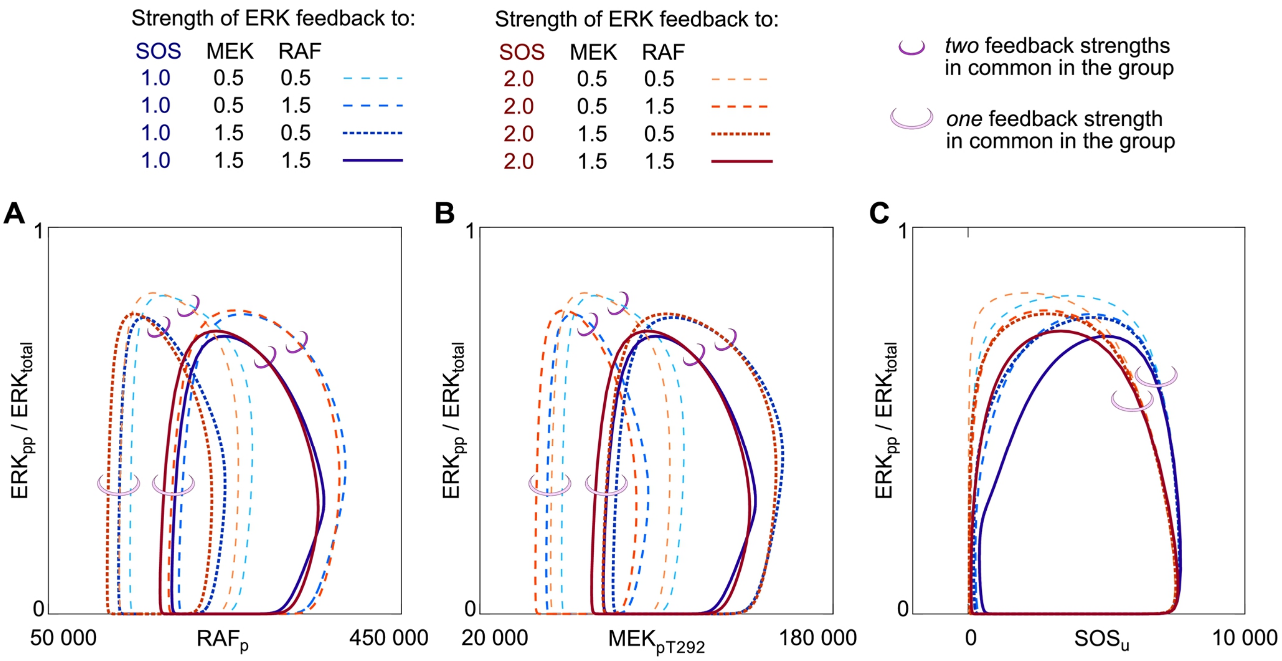

Supplementary Figure S4. Phase space analysis of sensitivity of oscillatory behavior to negative feedback strengths. Each diagram illustrates eight limit cycles in a different planar projection of phase space. As indicated in the legend at top by line style, each trajectory is associated with a unique set of strengths for the ERK-to-SOS, ERK-to-MEK, and ERK-to-RAF feedbacks. Blue curves indicate trajectories corresponding to the default strength of negative feedback from ERK to SOS; red curves indicate trajectories corresponding to a 2-fold higher strength. (**A**) Trajectories in the ERK_{pp}/ERK_{total}–RAF_p projection plane. (**B**) Trajectories in the ERK_{pp}/ERK_{total}–MEK_{pT292} projection plane. (**C**) Trajectories in the ERK_{pp}/ERK_{total}–SOS_u projection plane. These parametric plots indicate that trajectories cluster together depending on their associated feedback strengths. Tight clusters (involving trajectories having two feedback strengths in common) are decorated with small rings. Looser clusters (involving trajectories having a single feedback strength in common) are decorated with large rings. Importantly, clustering depends on projection plane.

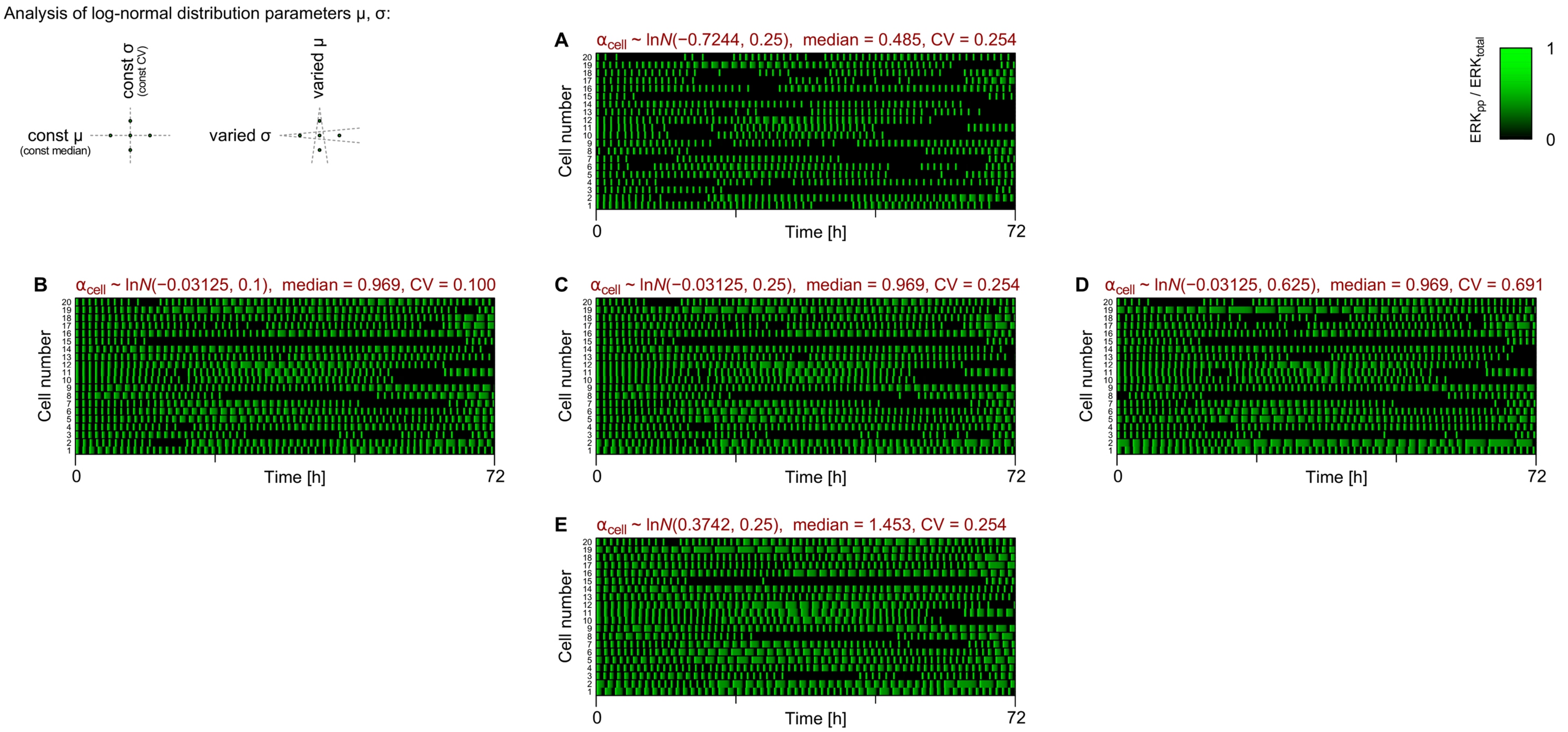

Supplementary Figure S5. Influence of extrinsic noise features on the oscillatory behavior. Each panel shows the results of 20 stochastic simulations of single-cell behavior for different values of two extrinsic noise parameters, which control the median and coefficient of variation (CV) of the EGFR surface expression burst size. Frequency of ERK activity pulses varies with the level of EGFR. In all simulations, oscillatory behavior is induced by an EGF dose of 10 pg/ml. Note that the central panel is for the same parameters as Fig. 8C (in the main text).

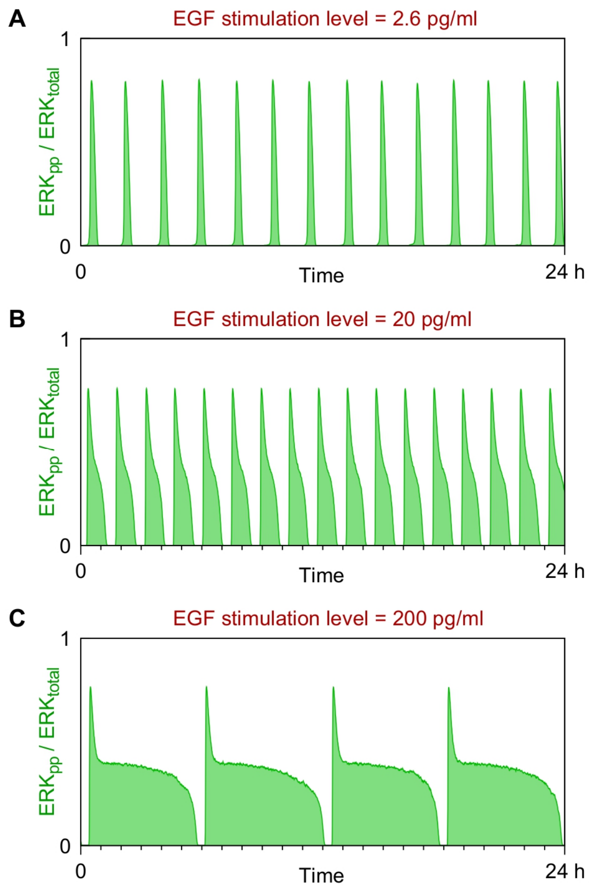

Supplementary Figure S6. Effect of intrinsic noise on system trajectories. At the assumed protein copy numbers per cell, the influence of intrinsic noise on system behavior is very weak; in panel C it can be visible as fine ripples. The simulations were performed using the Gillespie algorithm for default system parameters (Supplementary Table S1). This figure corresponds to Fig. 4 in the main text.

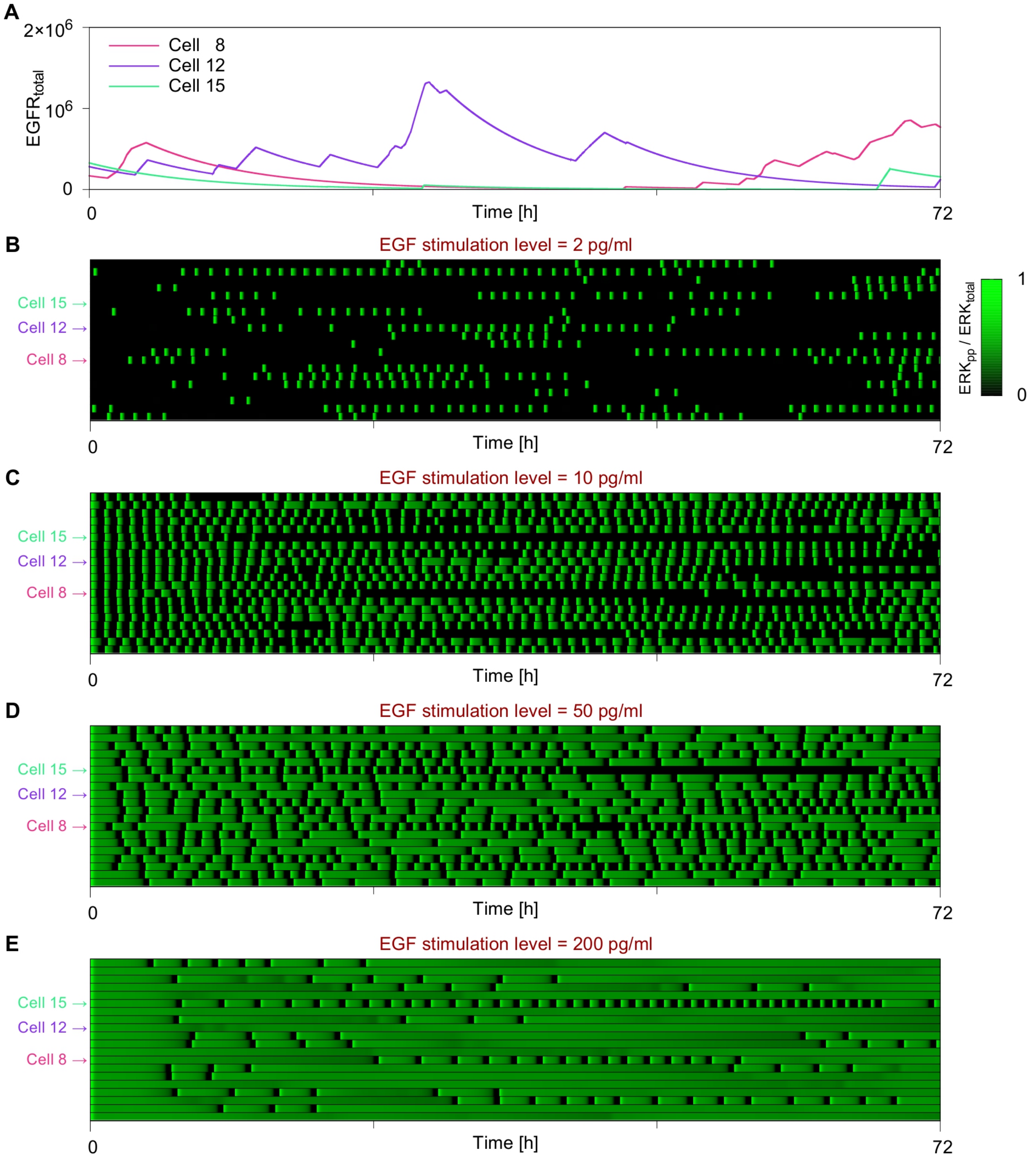

Supplementary Figure S7. Effect of extrinsic noise on system trajectories. The trajectories were obtained by piece-wise ODE integration. As can be seen by comparing this figure with Fig. 8 in the main text, the visible variability in cellular trajectories is predominantly due to the extrinsic noise.

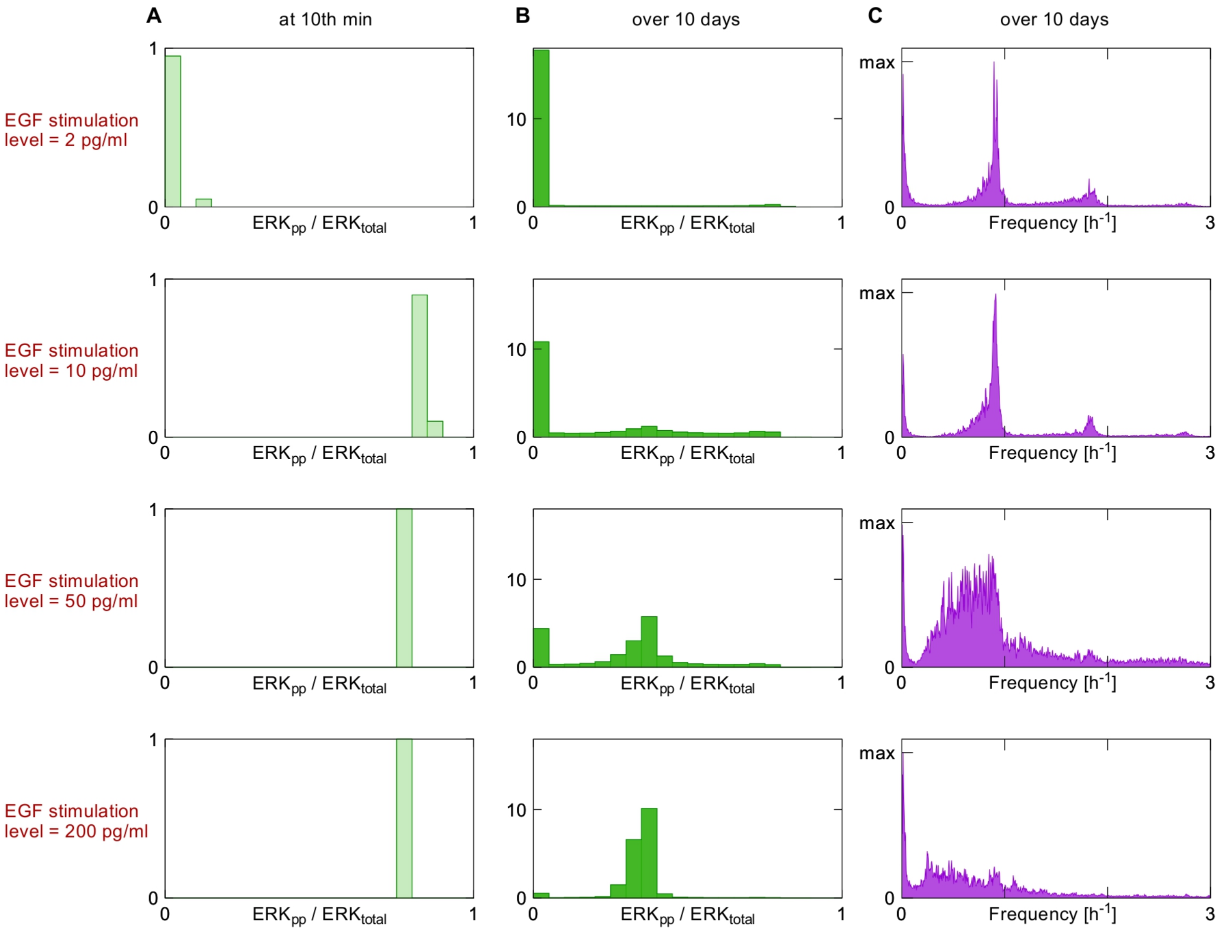

Supplementary Figure S8. Distributions of active ERK and its frequency spectrum. (**A**) Histograms of active ERK fractions at 10th min of EGF stimulation in simulations of 20 cells shown in panels of Fig. 8 in the main text. (**B**) Distributions of active ERK fractions over 10-day-long simulations of 20 cells. (**C**) Frequency spectrum of active ERK calculated from 10-day-long simulations of 20 cells.

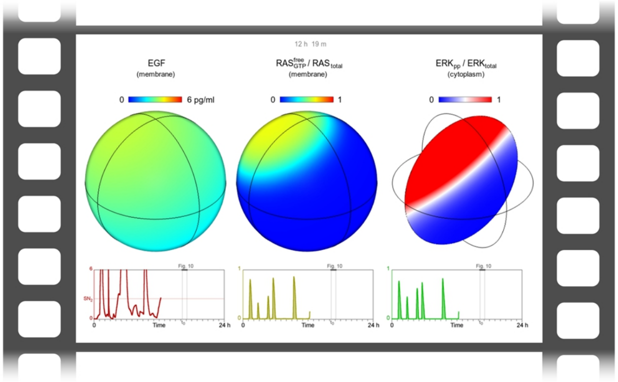

Video. Animation of COMSOL output. This video is provided as a separate MOV file. Activation of EGF receptors and RAS on the membrane, and activation of ERK in the cytoplasm (a cross-section is shown) in response to extracellular EGF bursts in the spatially extended, PDE-based model variant. Corresponding model source files are contained within Supplementary Dataset 4
